# Supplementary figures and images for: Effectiveness of Pseudomonas aeruginosa type VI secretion system relies on toxin potency and type IV pili-dependent interaction
Source: PLoS Pathog. 2023 May 30;19(5):e1011428. doi: 10.1371/journal.ppat.1011428 (PMC10281587; doi:10.1371/journal.ppat.1011428)

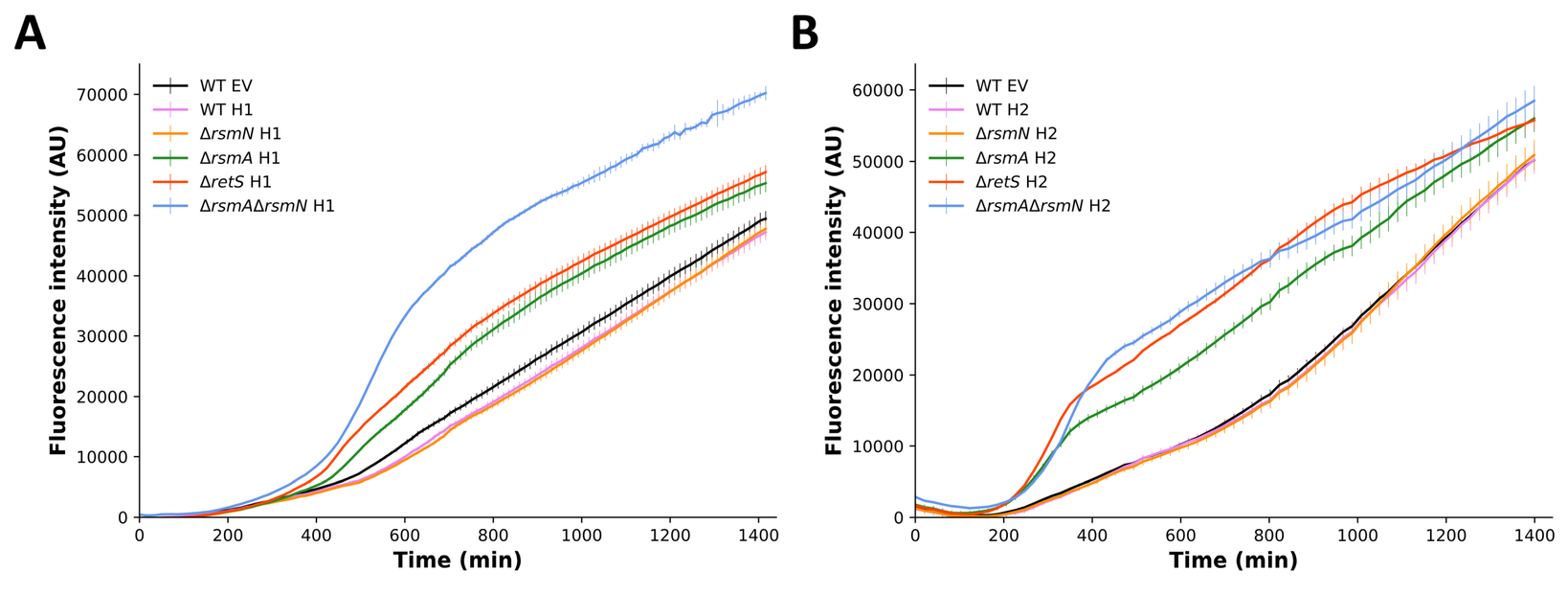

Supplement: S1 Fig — Analysis of H1-T6SS (A) and H2-T6SS (B) promoter translational activity in WT, ΔrsmN, ΔrsmA, ΔretS, and ΔrsmAΔrsmN strains. Measurements performed in static biofilm using plasmid based GFPmut3b reporter fusions. All measurements performed at 37°C, in static culture, each display item shows a mean +SD of 4 technical replicates that is representative of n = 3 biologically independent repeats. (TIF) [file ppat.1011428.s001.tif]

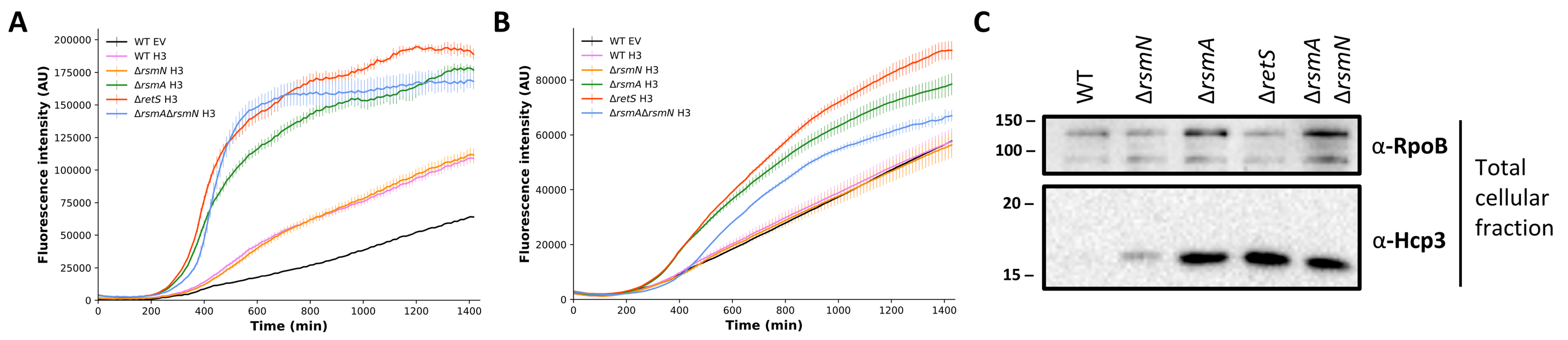

Supplement: S2 Fig — Analysis of H3-T6SS promoter transcriptional (A) and translational (B) activity in WT, ΔrsmN, ΔrsmA, ΔretS, and ΔrsmAΔrsmN strains. Measurements performed in static biofilm using plasmid based GFPmut3b reporter fusions. All measurements performed at 37°C, in static culture, each display item shows a mean +SD of 4 technical replicates that is representative of n = 3 biologically independent repeats. (C) Western blot analysis shows gradual elevation in of Hcp3 expression in WT, ΔrsmN, ΔrsmA, ΔretS, and ΔrsmAΔrsmN strains. A representative blot of 3 independent biological repeats shown here, for H3-T6SS activity assessment bacteria were cultured for 24h at 30°C. RNA polymerase (RpoB) used as loading control. (TIF) [file ppat.1011428.s002.tif]

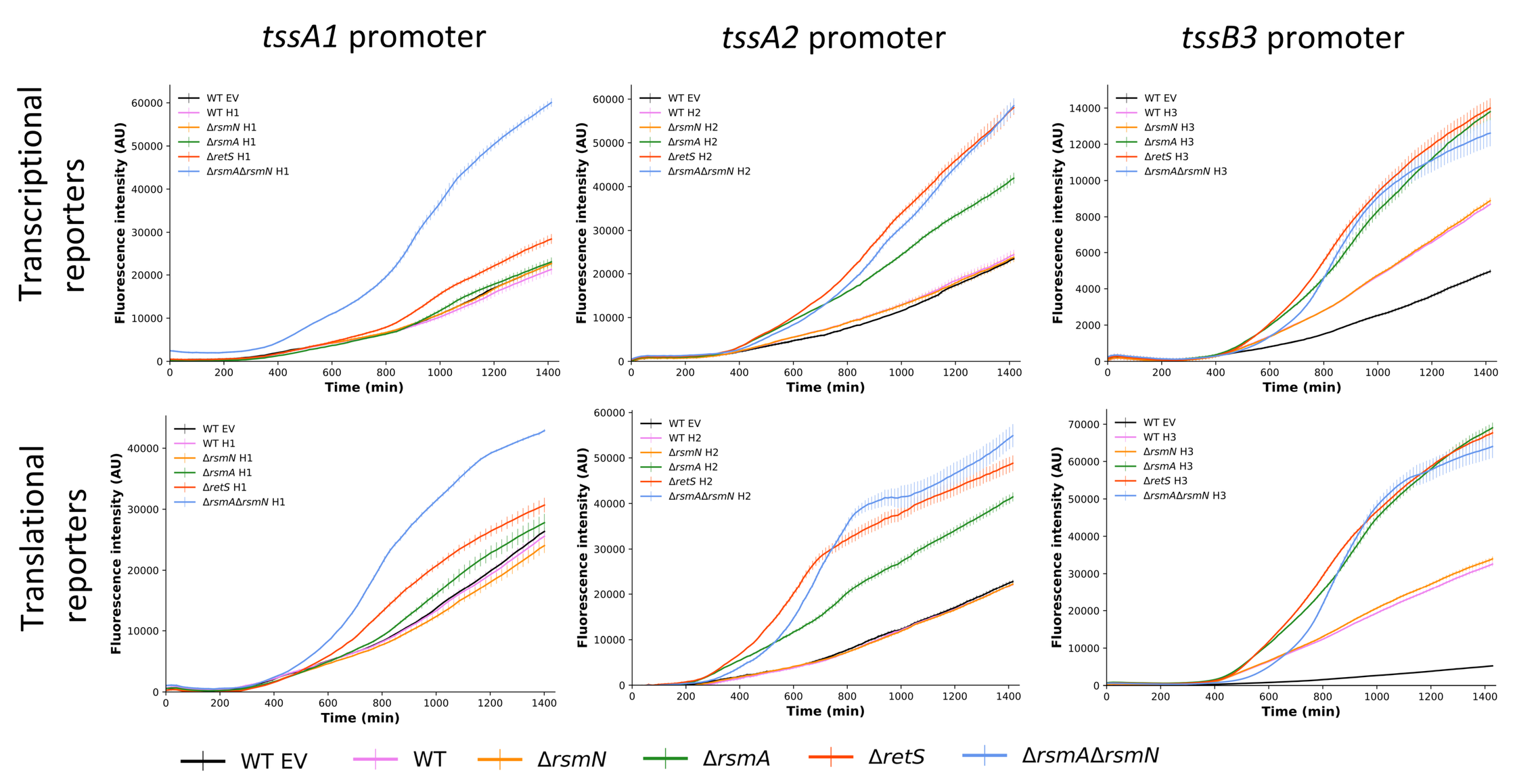

Supplement: S3 Fig — Analysis of T6SS promoter transcriptional (left) and translational (right) activity in WT, ΔrsmN, ΔrsmA, ΔretS, and ΔrsmAΔrsmN strains. Upper row shows H1-T6SS (tssA1), middle H2-T6SS (tssA2), and lower H3-T6SS (tssB3), promoter activity over growth time as measured by plasmid based GFPmut3b reporter fusions. All measurements performed at 25°C, in static culture, each display item shows a mean +SD of 4 technical replicates that is representative of n = 3 biologically independent repeats. (TIF) [file ppat.1011428.s003.tif]

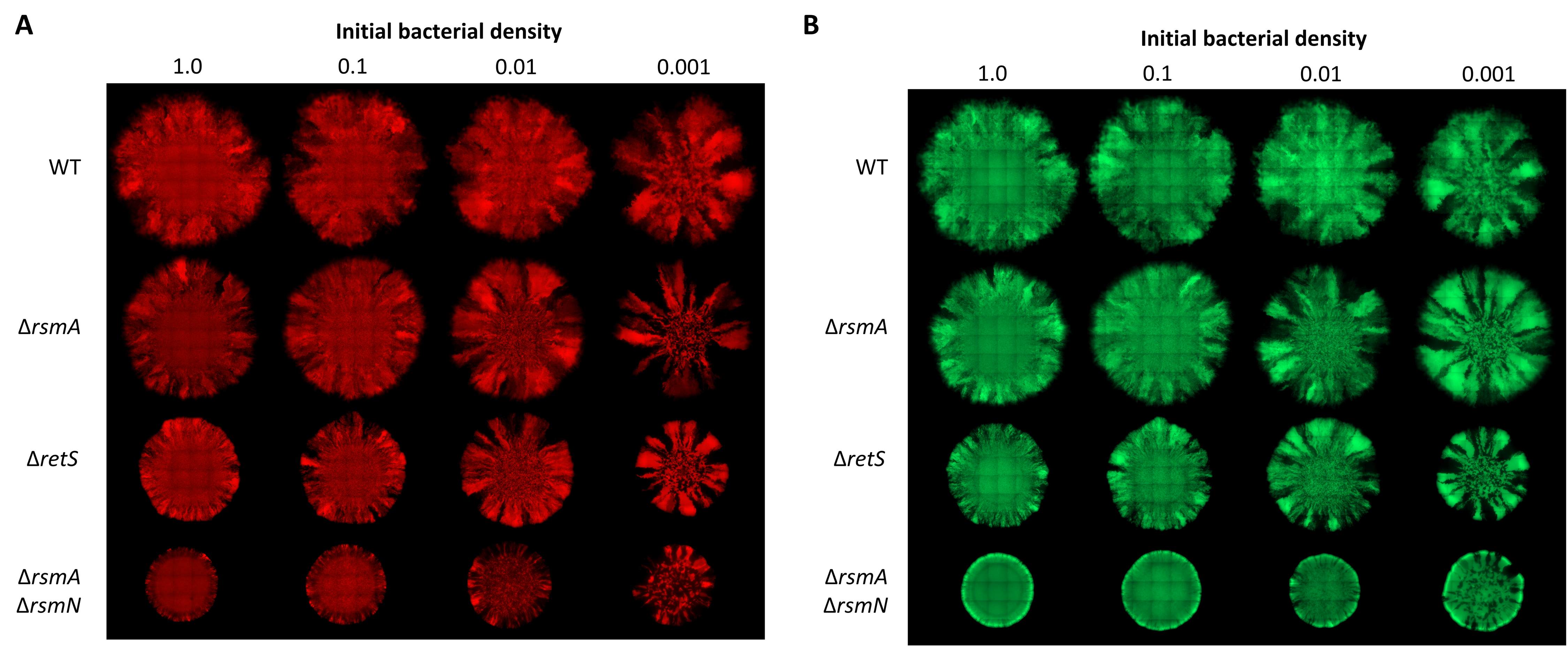

Supplement: S4 Fig — Single channel images corresponding to composite in Fig 2A–mCherry in red (A) and sfGFP in green (B). Mixed bacterial colonies of WT, ΔrsmA, ΔretS, and ΔrsmAΔrsmN strains with altered inoculum densities. Isogenic bacterial strains tagged with mCherry (red) and sfGFP (green) fluorophores were mixed at 1 to 1 ratio and after adjusting inoculum density (OD600 = 1.0; 0.1; 0.01; 0.001) spotted on LB agar, images of whole microcolonies taken after 48h incubation at 37°C show 2 morphologically distinct regions—highly mixed inner region corresponding to inoculum zone and outer region where spatial segregation of the sub-populations is apparent. (TIF) [file ppat.1011428.s004.tif]

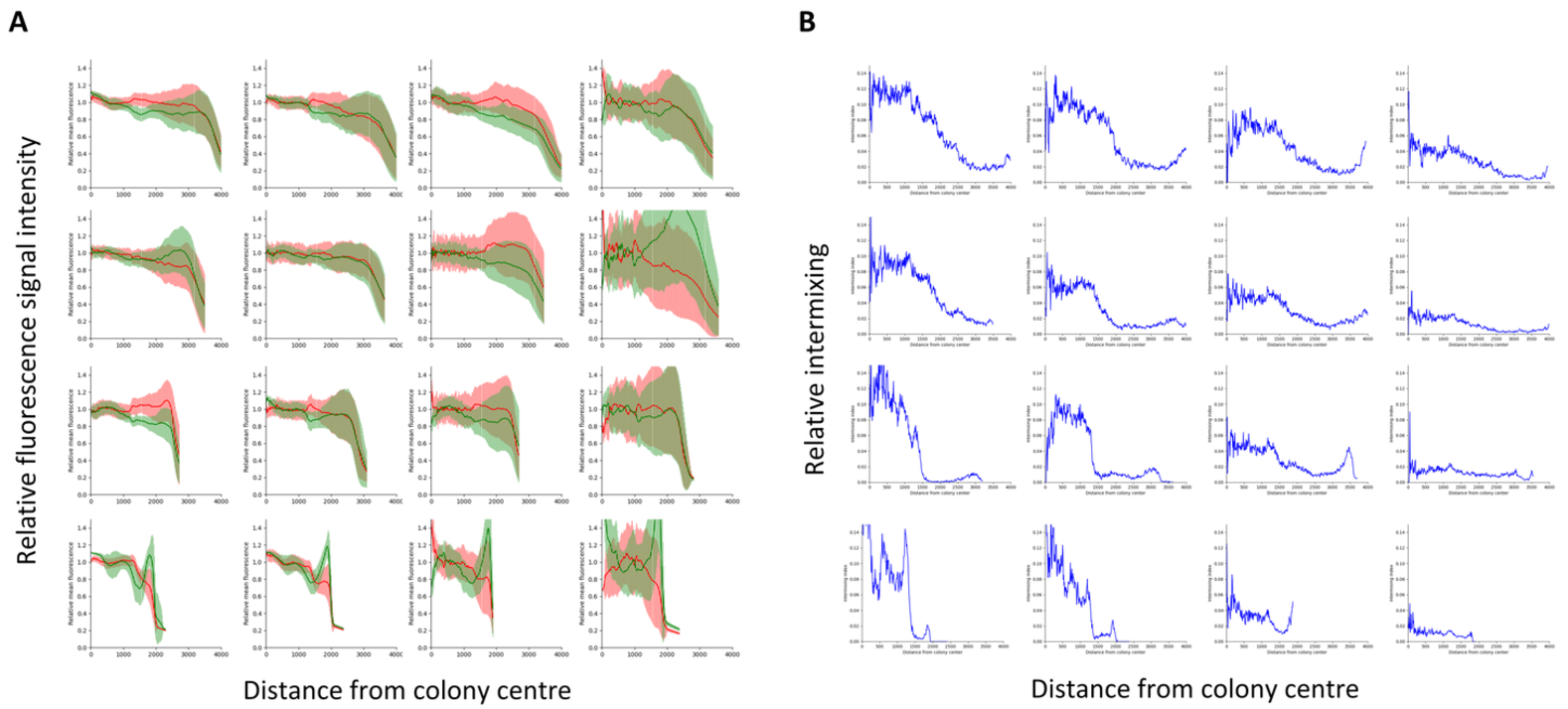

Supplement: S5 Fig — Analysis of spatial fluorescent signal distribution for fluorescence images shown in Fig 2. (A) Relative mean signal intensity + SD of individual fluorescence channels at different distances from the colony centre. (B) Relative sub-population intermixing at different distances from the macrocolony centre, calculated for circular sections taken at increasing distance from colony central point. (TIF) [file ppat.1011428.s005.tif]

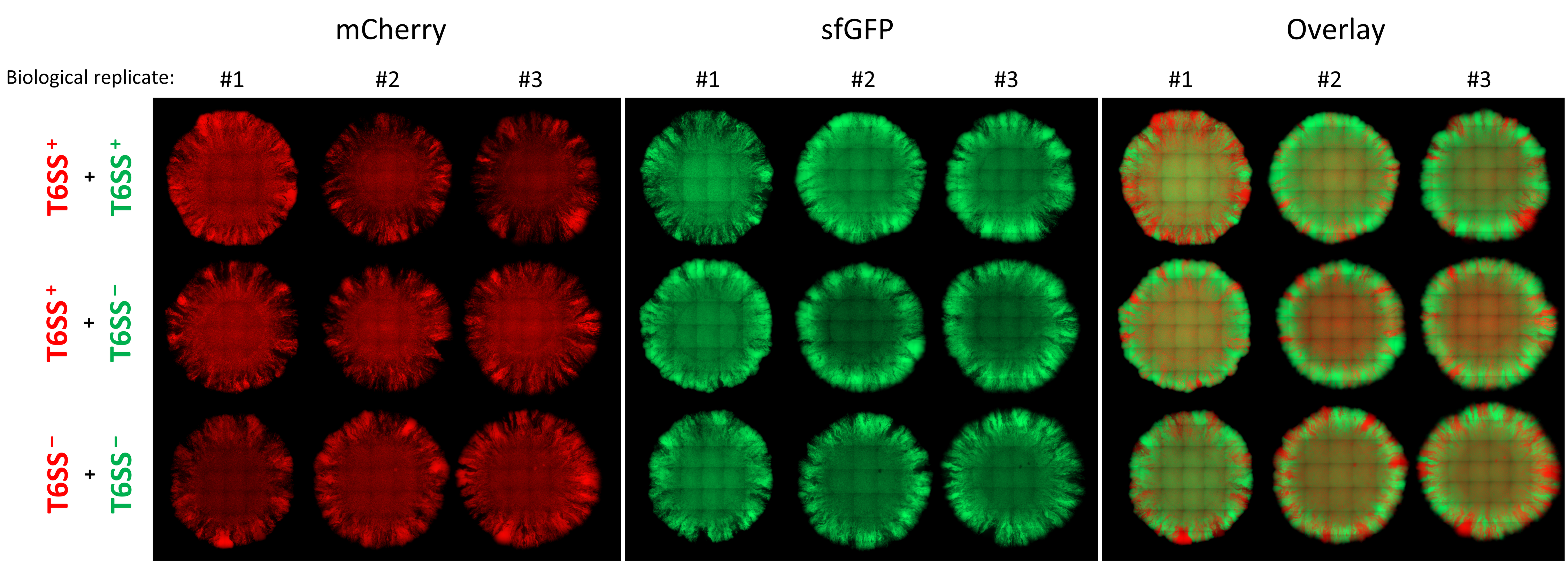

Supplement: S6 Fig — Fluorescent images of whole bacterial colonies of bacteria mixes made up of T6SS+ (ΔretS) and T6SS–(ΔretSΔtssB1ΔtssB2ΔtssB3) strains at 1:1 initial ratio and initial OD600 = 1.0, showing individual channel and overlay images of 3 biological repeats. (TIF) [file ppat.1011428.s006.tif]

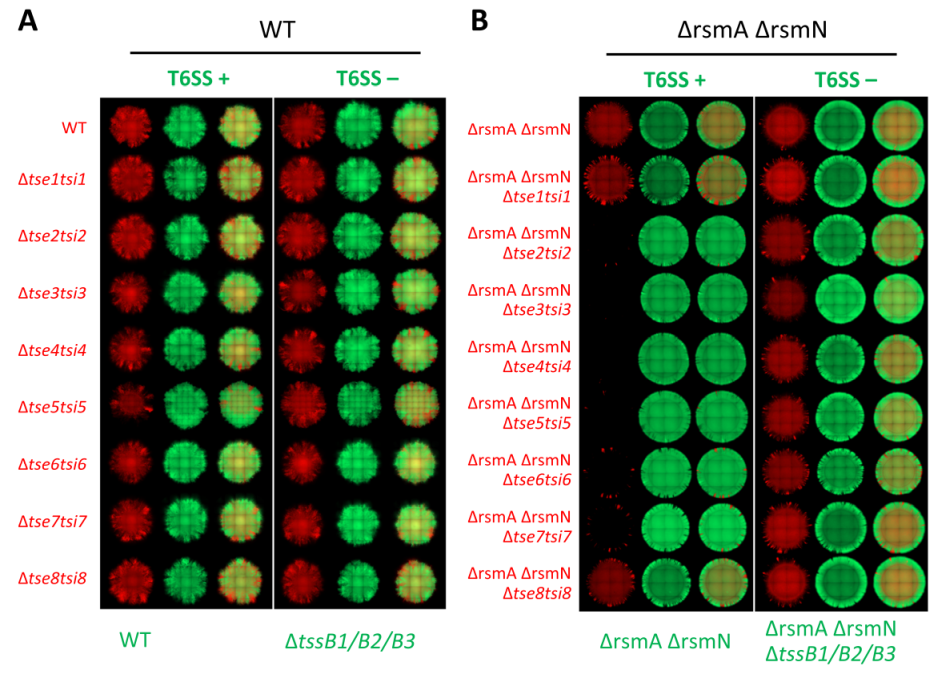

Supplement: S7 Fig — Representative image of 48h old mixed colonies of toxin sensitised bacteria in red in competition with T6SS+ or T6SS- (ΔtssB1ΔtssB2ΔtssB3) strains of the same regulatory background in green. Upper lane contains a control mix of bacteria with full toxin-immunity gene sets, each of the following lanes contains strain sensitised to one of the H1-T6SS toxins from Tse1 to Tse8. Images sets of competitions of WT(A) and ΔrsmAΔrsmN (B) background strains shown with each of the sets containing both single fluorescence channel and overlay images showing distribution of sensitised prey in a mix with T6SS+ and subsequently T6SS- (ΔtssB1ΔtssB2ΔtssB3) parental strain. Strains contain constitutively expressed fluorescent proteins, prey labelled with mCherry (shown in red) and attacker with sfGFP (shown in green). All bacteria mixed at 1:1 ratio, inoculum OD600 = 1.0, grown for 48h at 37°C on LB with 2% (w/v) agar. (TIF) [file ppat.1011428.s007.tif]

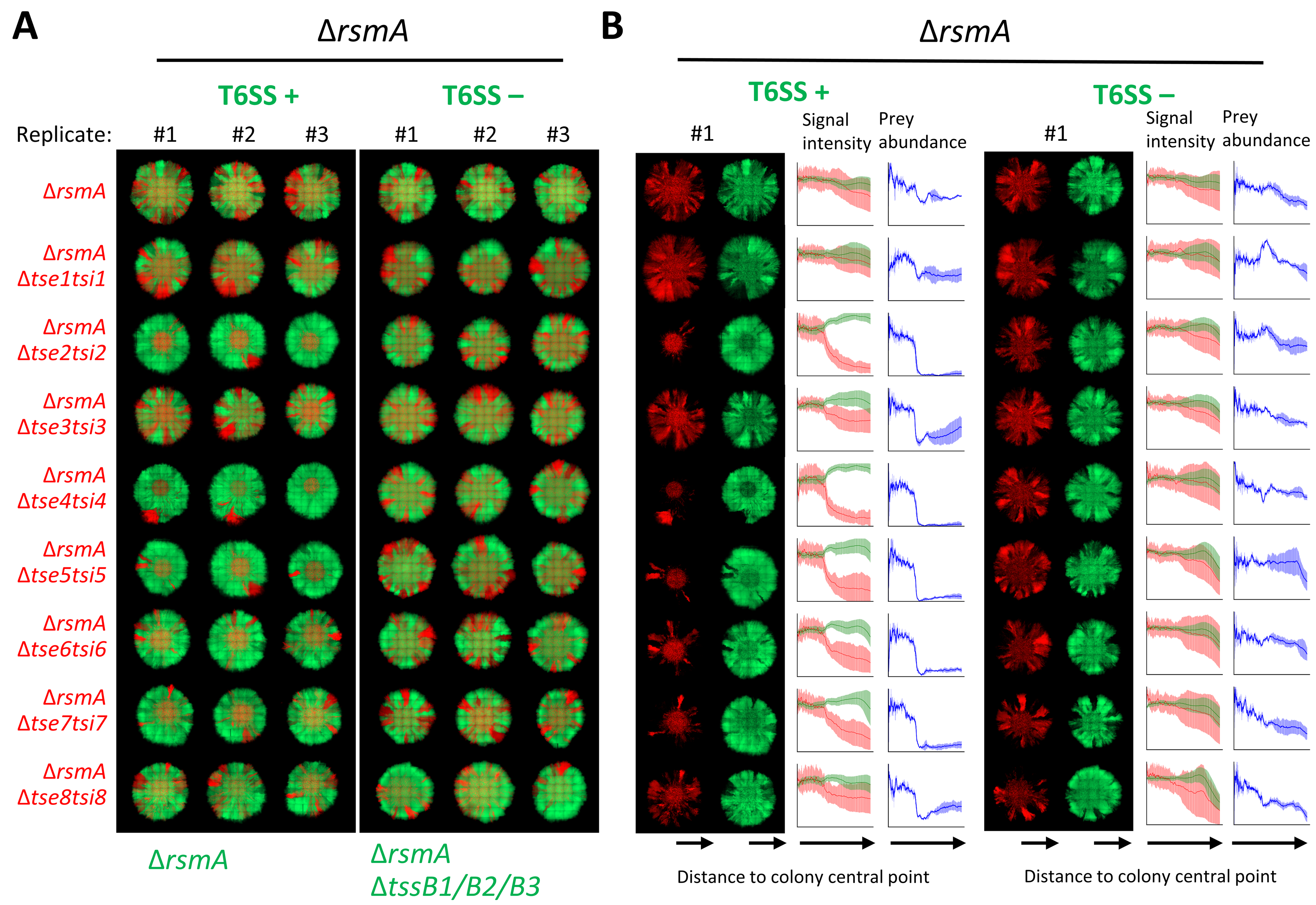

Supplement: S8 Fig — (A) Representative images of mixed bacterial macrocolonies consisting of H1-T6SS toxin sensitised bacteria (in red) in presence of T6SS+ (ΔrsmA) or T6SS- (ΔrsmAΔtssB1ΔtssB2ΔtssB3) parental bacteria (in green). Upper lane contains a control mix of bacteria with full toxin-immunity gene sets, each of the following lanes contains strain sensitised to one of the H1-T6SS toxins from Tse1 to Tse8. Fluorescent channel overlay images show bacterial distribution within 3 replicate colonies. All bacteria mixed at 1 to 1 ratio with inoculum density of OD600 = 0.01, grown for 48h at 37°C on LB with 2% (w/v) agar. (B) Images showing distribution of individual strains corresponding to replicates #1 from panel (A) and the corresponding analysis of fluorescent signal distribution within the image. Columns 2 and 5 show the corresponding mean (+SD) relative fluorescence signal intensity for the each of the channels as function of distance from the colony central point (for image replicates #1). With columns 3 and 6 showing relative area occupied by prey as function of distance from the colony central point (mean of 3 replicate colonies +SD). (TIF) [file ppat.1011428.s008.tif]

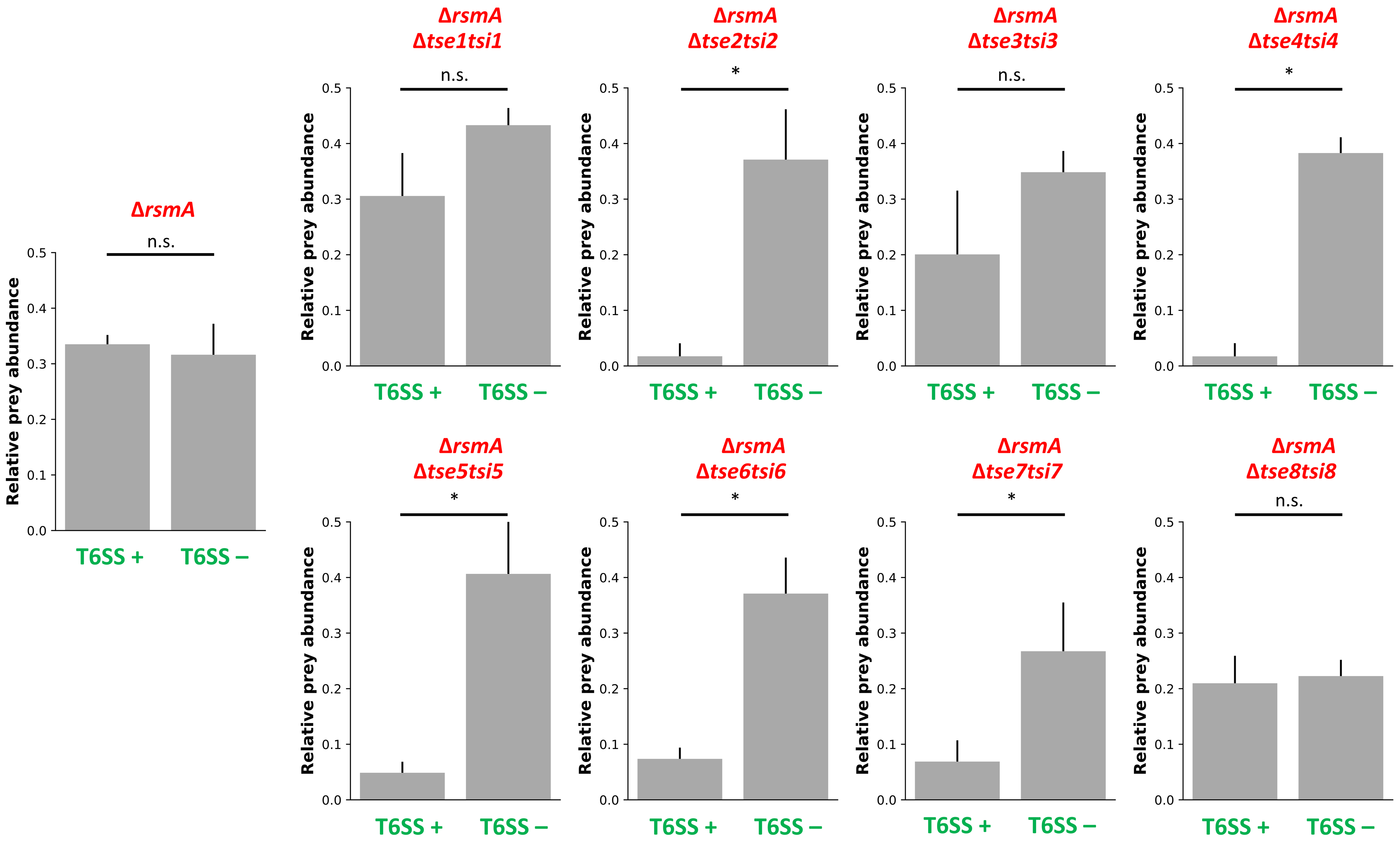

Supplement: S9 Fig — Summary analysis of images from S8 Fig, Measurement of relative outer colony region occupied by toxin sensitised prey in presence of T6SS+ (ΔrsmA) or T6SS- (ΔrsmAΔtssB1ΔtssB2ΔtssB3) parental bacteria. Mean (+SD) of 3 biological replicates, statistical significance measured using students T-test, where p<0.05 indicated with a “*”. P-values for each of the prey strains as follows– ΔrsmA– 0.6238; ΔrsmAΔtse1tsi1–0.0885; ΔrsmAΔtse2tsi2–0.0163; ΔrsmAΔts31tsi3–0.1450; ΔrsmAΔtse4tsi4–0.0009; ΔrsmAΔtse5tsi5–0.0474; ΔrsmAΔtse6tsi6–0.0101; ΔrsmAΔtse7tsi7–0.0429; ΔrsmAΔtse8tsi8–0.7214. (TIF) [file ppat.1011428.s009.tif]

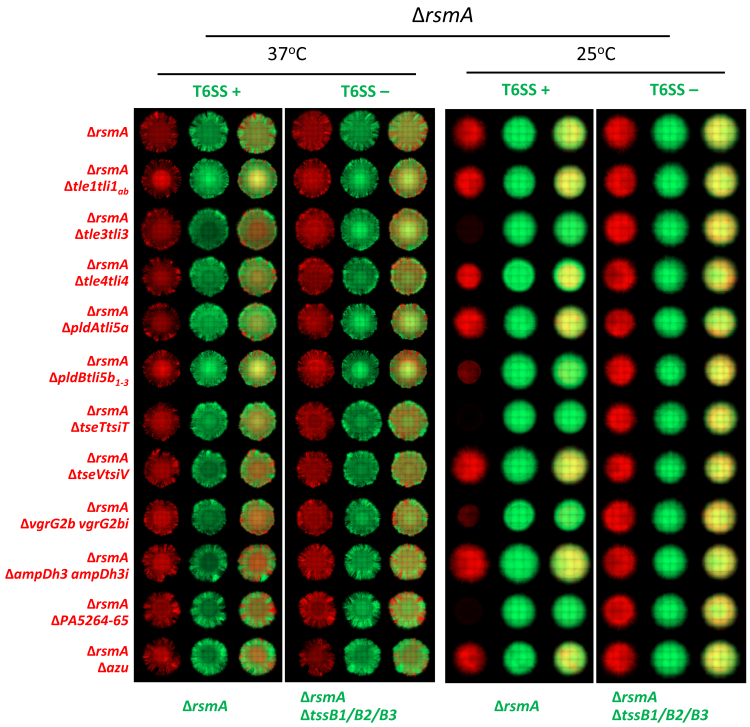

Supplement: S10 Fig — Sensitivity to H2-T6SS toxins can be observed when growing colonies at 25°C, but not 37°C. Representative images of 48h old mixed colonies of toxin sensitised ΔrsmA bacteria in red in competition with T6SS+ (ΔrsmA) or T6SS- (ΔrsmAΔtssB1ΔtssB2ΔtssB3) parental strain in green. Upper lane contains a control mix of bacteria with full toxin-immunity gene sets, each of the following lanes contains strain sensitised to one of the H2-T6SS toxins in the following order: Tle1, Tle3, Tle4, PldA, PldB, TseT, TseV, VrgG2b, AmpDh3, PA5265, and common good effector Azu. Image sets of competitions show both single fluorescence channel and overlay images depicting distribution of sensitised prey in a mix with T6SS+ and subsequently T6SS- (ΔtssB1ΔtssB2ΔtssB3) parental strains at 2 different growth temperatures– 37°C and 25°C. Strains contain constitutively expressed fluorescent proteins, prey labelled with mCherry (shown in red) and attacker with sfGFP (shown in green). All bacteria mixed at 1:1 ratio, inoculum OD600 = 1.0, grown for 48h, at 37°C bacteria incubated on LB with 2% (w/v) agar, and at 25°C bacteria incubated on LB with 1.2% (w/v) agar. (TIF) [file ppat.1011428.s010.tif]

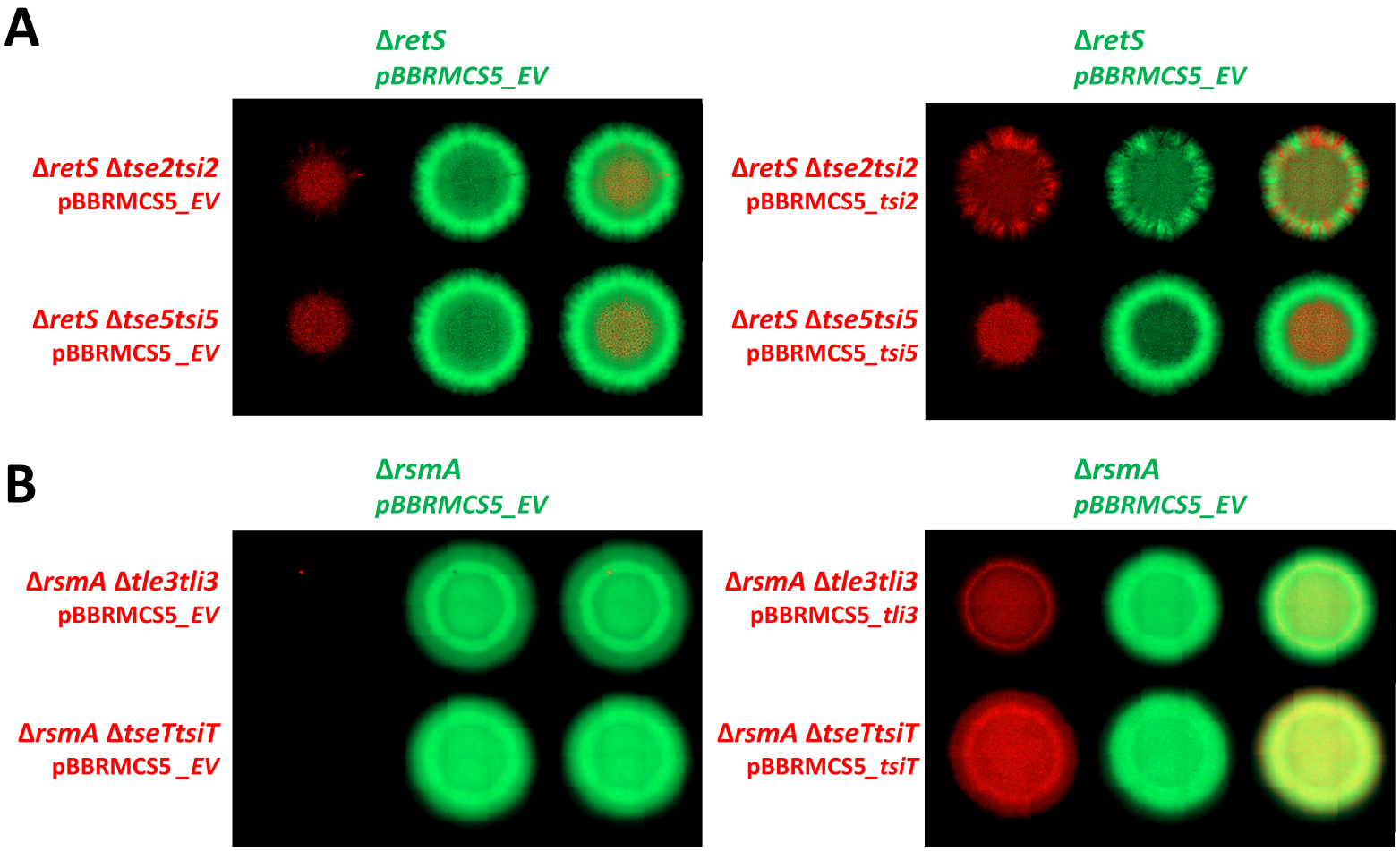

Supplement: S11 Fig — Representative mixed colony images showing protective impact of toxin immunity complementation in both H1-T6SS (A) and H2-T6SS (B) toxin-dependent competition. Showing individual fluorescence channel and overlay images of toxin sensitised prey (in red) carrying empty vector plasmid (left) and a plasmid expressing cognate immunity. (A) Tse2 (ΔretSΔtse2tsi2) and Tse5 (ΔretSΔtse5tsi5) sensitive prey bacteria in competition with T6SS+ (ΔretS) parental strain. Bacteria mixed at 1 to 1 ratio, with inoculum density OD600 = 1.0, grown for 48h at 37°C on LB with 2% (w/v) agar. (B) Tle3 (ΔrsmAΔtle3tli3) and TseT (ΔrsmAΔtseTtsiT) sensitised prey bacteria in a mix with T6SS+ (ΔrsmA) parental strain. Bacteria mixed at 1 to 1 ratio, with inoculum density OD600 = 1.0, grown for 48h at 25°C on LB with 1.2% (w/v) agar. Individual images of 3 biological replicates shown. (TIF) [file ppat.1011428.s011.tif]

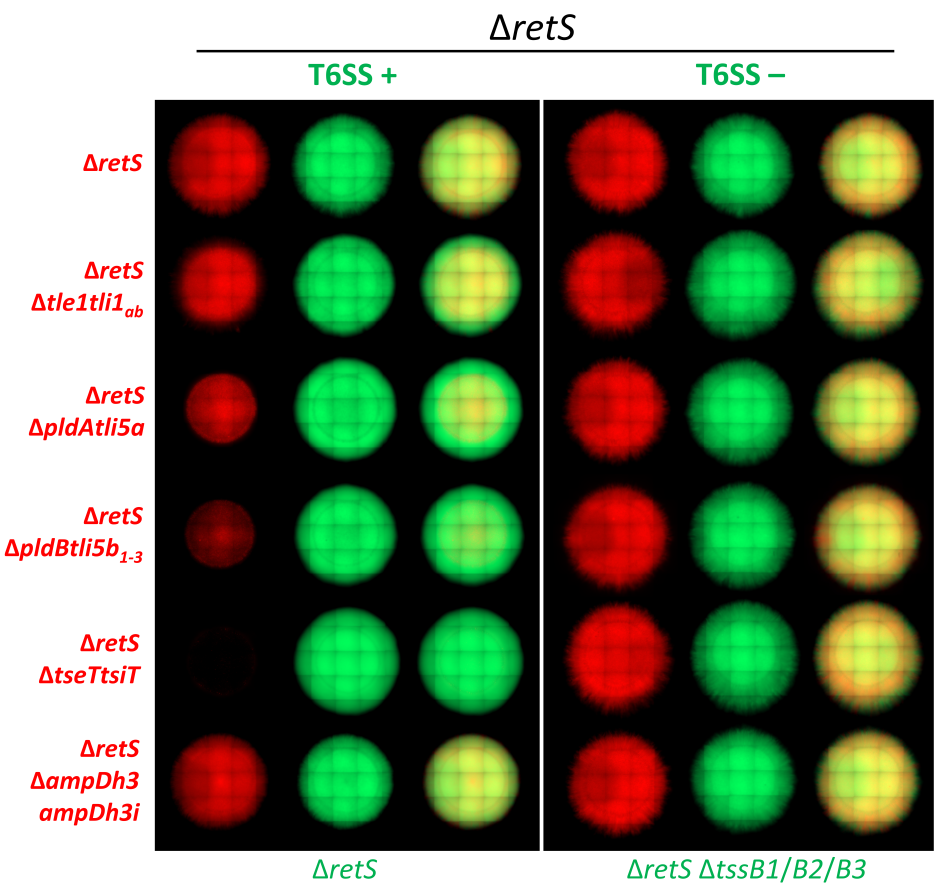

Supplement: S12 Fig — Representative images of 48h old mixed colonies of toxin sensitised ΔretS bacteria in red in competition with T6SS+ (ΔretS) or T6SS- (ΔretSΔtssB1ΔtssB2ΔtssB3) parental strain in green. Upper lane contains a control mix of bacteria with full toxin-immunity gene sets, each of the following lanes contains strain sensitised to one of the H2-T6SS toxins in the following order: Tle1, PldA, PldB, TseT and AmpDh3. Image sets of competitions show both single fluorescence channel and overlay images depicting distribution of sensitised prey in a mix with T6SS+ and subsequently T6SS- (ΔtssB1ΔtssB2ΔtssB3) parental strains. Strains contain constitutively expressed fluorescent proteins, prey labelled with mCherry (shown in red) and attacker with sfGFP (shown in green). All bacteria mixed at 1:1 ratio, inoculum OD600 = 1.0, grown for 48h at 25°C on LB with 1.2% (w/v) agar. (TIF) [file ppat.1011428.s012.tif]

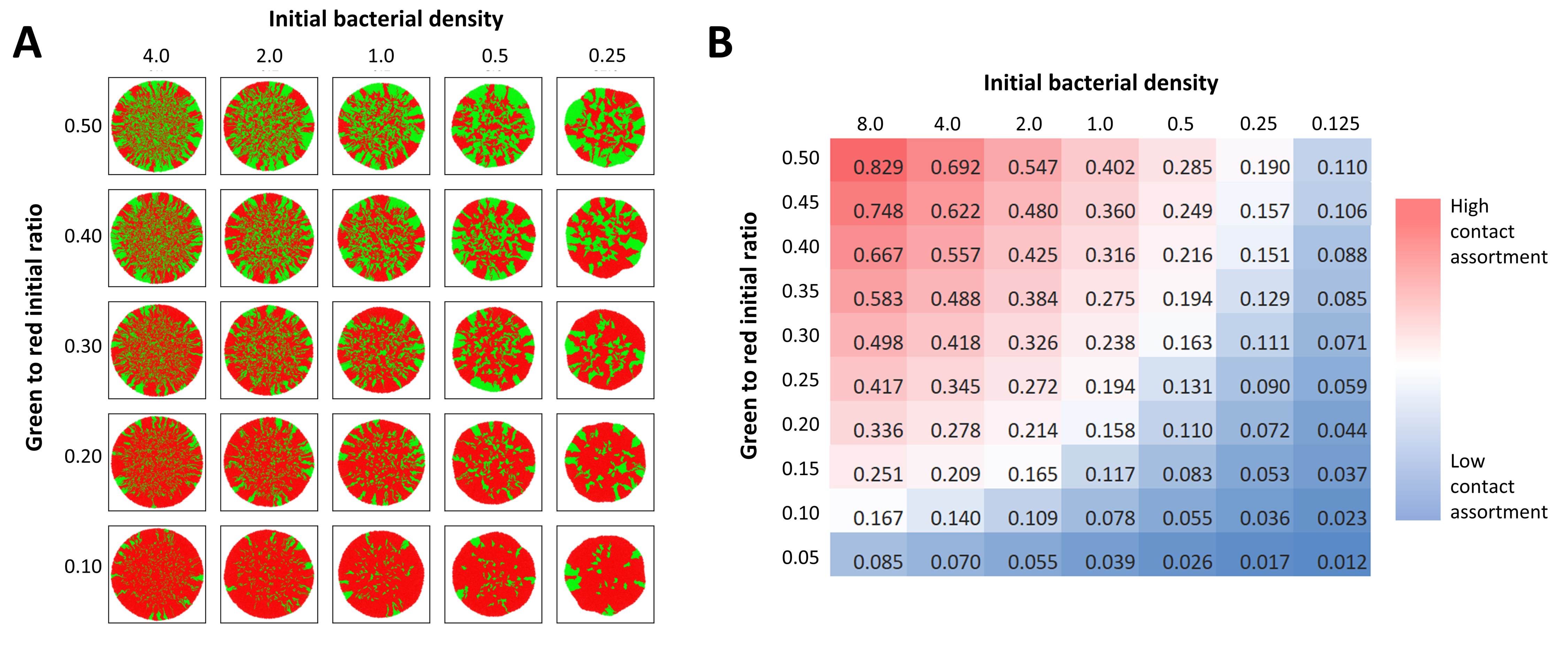

Supplement: S13 Fig — (A) Representative simulation outputs showing changes species distribution resulting from variation in initial density and mixing ratio of the populations. (B) Changes in localised agent intermixing as interspecies contact assortment resulting from variation in initial density and mixing ratio of cells in simulation setup. (No T6SS interactions, mean of n = 5). (TIF) [file ppat.1011428.s013.tif]

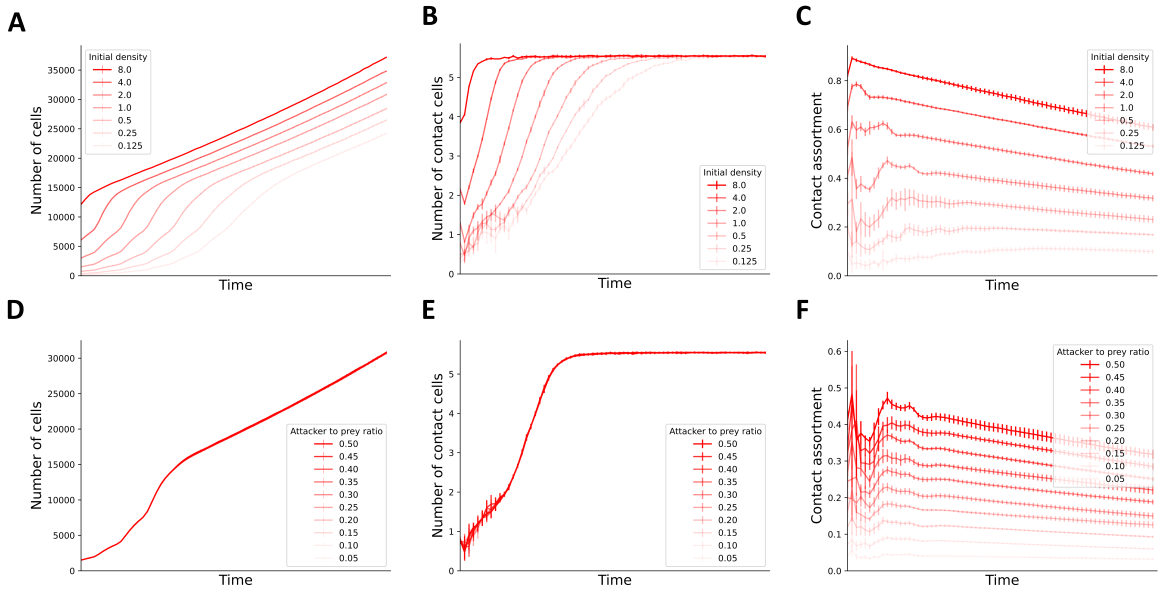

Supplement: S14 Fig — (A, D) Total number of cells over simulation time-course. (B, E) Total number of contact cells over simulation time-course. (C, F) Interspecies contact assortment over 30 simulation time-course. A, B, and C effect of variation in initial density. (D, E, F) effect of variation in species mixing ratio. (No T6SS interactions, mean +SD of n = 5). (TIF) [file ppat.1011428.s014.TIF]

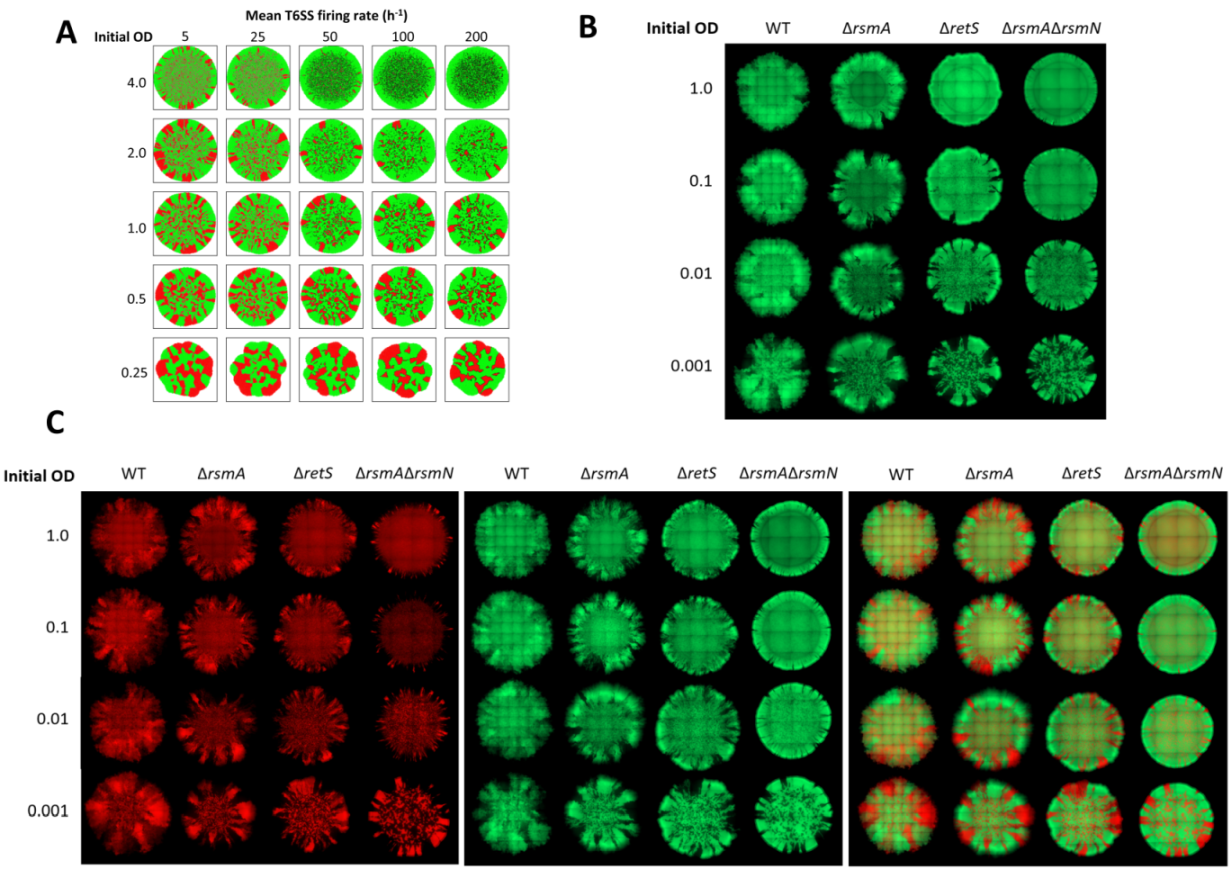

Supplement: S15 Fig — Set of simulation outputs (A) corresponding to Fig 5C with non-lytic toxin-based interactions. Set of corresponding green-fluorescent channel images (B) for the Fig 5D and a set of images of preys from Fig 5D in a mix with an T6SS- attacker strain (C). (A) Representative simulation outputs showing how decrease in initial bacterial density promotes prey (red) survival in a mix an attacker population (green) with differing mean T6SS firing rate within a context of non-lytic toxins. (Toxin lethal dose = 5). (B) Single channel images corresponding to composite in Fig 5D showing distribution of attacker population in green. (C) Single channel and composite images of the prey set from Fig 5D. In a mix with T6SS- attacker (ΔtssB1ΔtssB2ΔtssB3) of corresponding regulatory background. Mixed bacterial colonies of WT, ΔrsmA, ΔretS, and ΔrsmAΔrsmN strains with altered inoculum densities. Isogenic bacterial strains tagged with mCherry (red) and sfGFP (green) fluorophores were mixed at 1 to 1 ratio and after adjusting inoculum density (OD = 1.0; 0.1; 0.01; 0.001) spotted on LB agar, images of whole microcolonies taken after 48h incubation at 37°C on LB with (2% w/v) agar. (TIF) [file ppat.1011428.s015.tif]

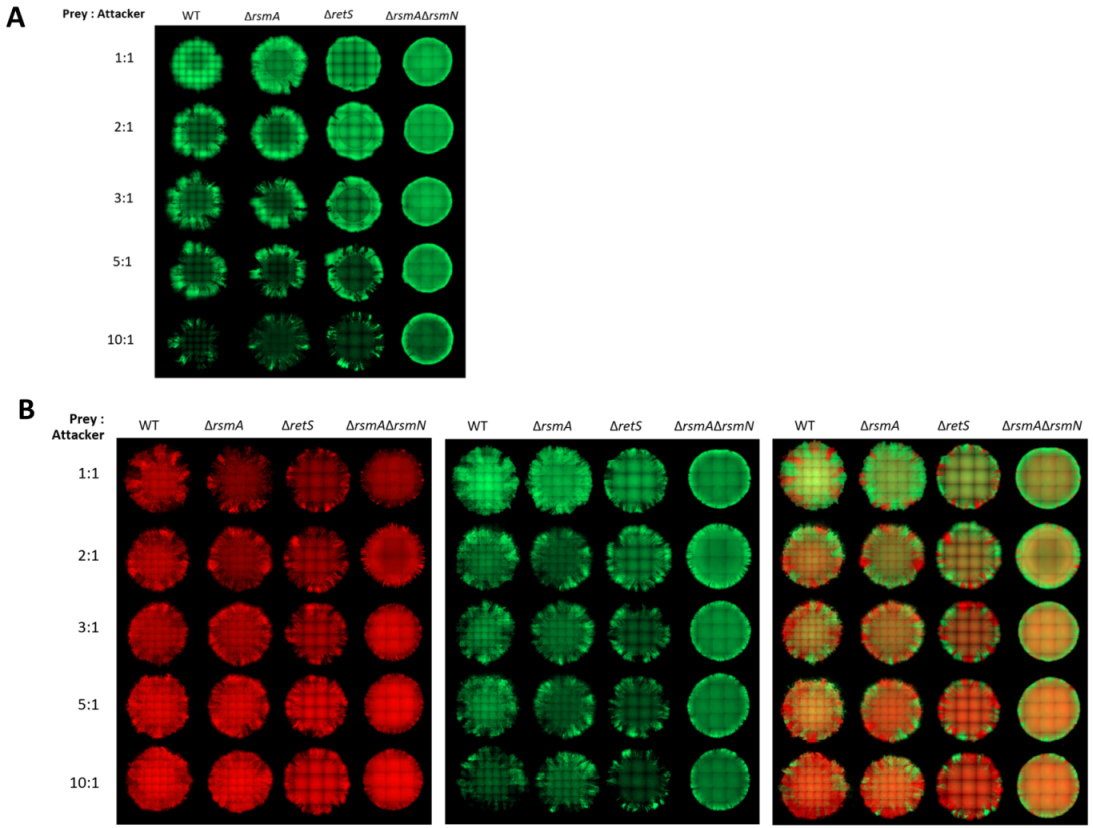

Supplement: S16 Fig — Set of corresponding green-fluorescent channel images (A) for the Fig 5E and a set of images of preys from Fig 5E in a mix with an T6SS- attacker strain (B). (A) Fluorescent single (sfGFP) channel images of mixed colonies from Fig 5F showing distribution of T6SS+ attacker strains. (B) Tse5 sensitive prey spatial distribution in presence of T6SS- (ΔtssB1ΔtssB2ΔtssB3) parental competitor strain. Individual and overlaid channel images shown. Each of the columns correspond to WT, ΔrsmA, ΔretS, and ΔrsmAΔrsmN regulatory background strains in the given order. Each of the rows contains a set of representative images of colonies set up with a different prey to attacker ratio in the inoculum. Prey to attacker ratios from the top row are as follows: 1:1, 2:1, 3:1, 5:1, 10:1. (Strains contain constitutively expressed fluorescent proteins, prey labelled with mCherry (shown in red) and attacker with sfGFP (shown in green). All bacteria mixed at ratios specified, inoculum OD600 = 0.01, grown for 48h at 37°C on LB with 2% (w/v) agar. (TIF) [file ppat.1011428.s016.TIF]

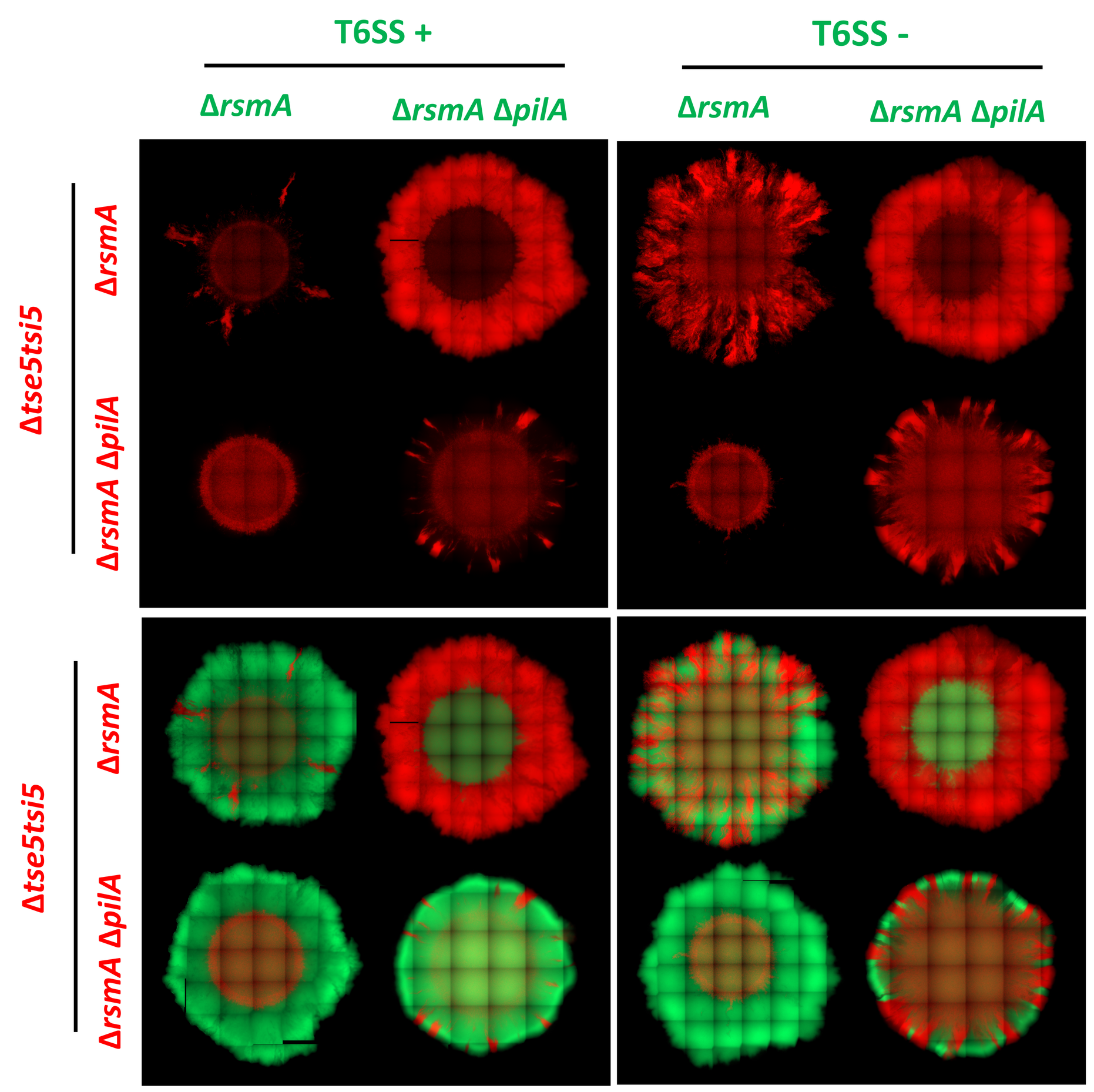

Supplement: S17 Fig — Tse5 mediated competition was used to assess impact on H1-T6SS mediated killing in a mix of T4P+ and T4P- bacteria. The panel contains individual red channel images showing toxin sensitive prey distribution in presence of T6SS+ (left) or T6SS- (right) attacker strain in the upper two rows. Corresponding overlay images showing distribution of both toxin sensitive prey (in red—mCherry) and T6SS attacker population in green (sfGFP) shown in the lower two rows. T4P+ prey (ΔrsmA Δtse5tsi5) strains in the upper row, with T4P- prey (ΔrsmA ΔpilA Δtse5tsi5) in the lower row. With T4P+ attacker in the first column and T4P- attacker strains in the second. All bacteria mixed at 1 to 1 ratio, with inoculum density OD600 = 1.0, grown for 48h at 37°C on LB with 2% (w/v) agar. 1 of 1 biological repeat shown for all images containing ΔpilA mutant strains. (TIF) [file ppat.1011428.s017.tif]

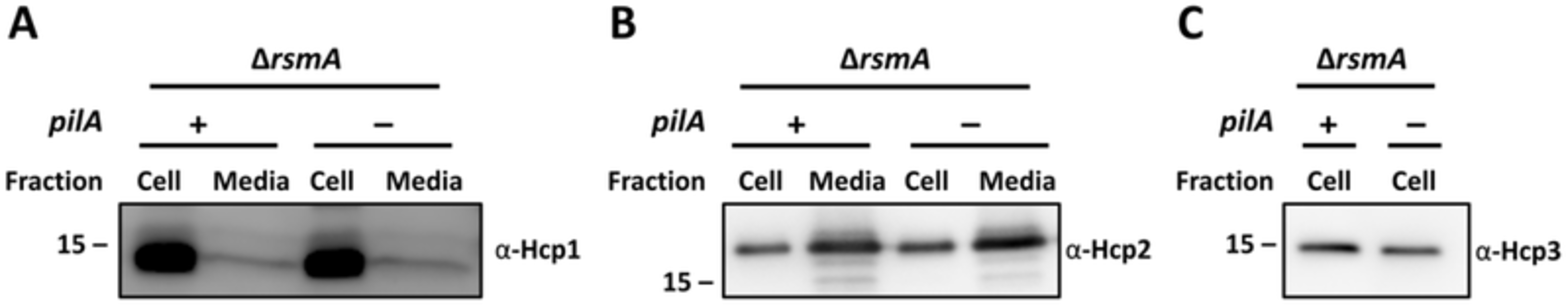

Supplement: S18 Fig — Western blot analysis shows that Hcp1(A), Hcp2 (B), and Hcp3 (C) protein levels remain consistent within cell lysate independently of the presence of T4P (ΔpilA). As assessed through secretion assay, no differences in H1-T6SS (A) and H2-T6SS (B) system activity are detectable upon loss of T4P. Bacteria were cultured for 5-6h at 37°C (H1-T6SS), for 8h at 25°C (H2-T6SS) and for 24h at 25°C (H3-T6SS). (TIF) [file ppat.1011428.s018.tif]
