# Supplementary material for: Effectiveness of Pseudomonas aeruginosa type VI secretion system relies on toxin potency and type IV pili-dependent interaction
Source: PLoS Pathog. 2023 May 30;19(5):e1011428. doi: 10.1371/journal.ppat.1011428 (PMC10281587; doi:10.1371/journal.ppat.1011428)
Supplement: S1 Table — (DOCX) [file ppat.1011428.s022.docx]

**S1 Table List of strains used in this study**

| **Strain** | **Features** | **Source** |
| --- | --- | --- |
| PAO1 | Wild type strain, parental strain used in construction of all other strains in this study | Laboratory collection |
| Δ*rsmN* | Deletion of *rsmN* (PA5183.1) | This study |
| Δ*rsmA* | Deletion of *rsmA* (PA0905) | Laboratory collection |
| Δ*retS* | Deletion of *retS* (PA4856) | Laboratory collection |
| Δ*rsmA*Δ*rsmN* | Deletion of *rsmA* (PA0905) and *rsmN* (PA5183.1) | This study |
| Δ*tssB1*Δ*tssB2*Δ*tssB3* | Deletion of *tssB1* (PA0083), *tssB2* (PA1657) and *tssB3* (PA2365) | Laboratory collection |
| Δ*rsmN*Δ*tssB1*Δ*tssB2*Δ*tssB3* | Deletion of *rsmN* (PA5183.1), *tssB1* (PA0083), *tssB2* (PA1657) and *tssB3* (PA2365) | This study |
| Δ*rsmA*Δ*tssB1*Δ*tssB2*Δ*tssB3* | Deletion of *rsmA* (PA0905), *tssB1* (PA0083), *tssB2* (PA1657) and *tssB3* (PA2365) | Laboratory collection |
| Δ*retS*Δ*tssB1*Δ*tssB2*Δ*tssB3* | Deletion of *retS* (PA4856), *tssB1* (PA0083), *tssB2* (PA1657) and *tssB3* (PA2365) | This study |
| Δ*rsmA*Δ*rsmN*Δ*tssB1*Δ*tssB2*ΔtssB3 | Deletion of *rsmA* (PA0905), *rsmN* (PA5183.1), *tssB1* (PA0083), *tssB2* (PA1657) and *tssB3* (PA2365) | This study |
| Δ*rsmA*::*tssB1-mScarlet-I* | Deletion of *rsmA* (PA0905), encoding C-terminal mScarlet-I fusion to TssB1 (PA0083) at native locus | Laboratory collection |
| Δ*retS*::*tssB1-mScarlet-I* | Deletion of *retS* (PA4856), encoding C-terminal mScarlet-I fusion to TssB1 (PA0083) at native locus | Laboratory collection |
| Δ*rsmA*Δ*rsmN*::*tssB1-mScarlet-I* | Deletion of *rsmA* (PA0905) and *rsmN* (PA5183.1), encoding C-terminal mScarlet-I fusion to TssB1 (PA0083) at native locus | This study |
| Δ*tse1_tsi1* | Deletion of *tse1* and *tsi1* (PA1844-45) | This study |
| Δ*tse2_tsi2* | Deletion of *tse2* and *tsi2* (PA2702-03) | This study |
| Δ*tse3_tsi3* | Deletion of *tse3* and *tsi3* (PA3484-85) | This study |
| Δ*tse4_tsi4* | Deletion of *tse4* and *tsi4* (PA2774-75) | This study |
| Δ*tse5_tsi5* | Deletion of *tse5* and *tsi5* (PA2683-84) | This study |
| Δ*tse6_tsi6* | Deletion of *tse6* and *tsi6* (PA1844-45) | This study |
| Δ*tse7_tsi7* | Deletion of *tse7* and *tsi7* (PA0099-0100) | This study |
| Δ*tse8_tsi8* | Deletion of *tse8* and *tsi8* (PA1844-45) | This study |
| Δ*rsmA*Δ*tse1_tsi1* | Deletion of *rsmA* (PA0905), *tse1* and *tsi1* (PA1844-45) | Laboratory collection |
| Δ*rsmA*Δ*tse2_tsi2* | Deletion of *rsmA* (PA0905), *tse2* and *tsi2* (PA2702-03) | Laboratory collection |
| Δ*rsmA*Δ*tse3_tsi3* | Deletion of *rsmA* (PA0905), *tse3* and *tsi3* (PA3484-85) | Laboratory collection |
| Δ*rsmA*Δ*tse4_tsi4* | Deletion of *rsmA* (PA0905), *tse4* and *tsi4* (PA2774-75) | This study |
| Δ*rsmA*Δ*tse5_tsi5* | Deletion of *rsmA* (PA0905), *tse5* and *tsi5* (PA2683-84) | This study |
| Δ*rsmA*Δ*tse6_tsi6* | Deletion of *rsmA* (PA0905), *tse6* and *tsi6* (PA0092-93) | This study |
| Δ*rsmA*Δ*tse7_tsi7* | Deletion of *rsmA* (PA0905), *tse7* and *tsi7* (PA0099-0100) | This study |
| Δ*rsmA*Δ*tse8_tsi8* | Deletion of *rsmA* (PA0905), *tse8* and *tsi8* (PA1844-45) | This study |
| Δ*rsmA*Δ*tse1_tsi1*Δ*tse3_tsi3* | Deletion of *rsmA* (PA0905), *tse1* and *tsi1* (PA1844-45), *tse3* and *tsi3* (PA3484-85) | Laboratory collection |
| Δ*retS*Δ*tse1_tsi1* | Deletion of *retS* (PA4856), *tse1* and *tsi1* (PA1844-45) | This study |
| Δ*retS*Δ*tse2_tsi2* | Deletion of *retS* (PA4856), *tse2* and *tsi2* (PA2702-03) | This study |
| Δ*retS*Δ*tse3_tsi3* | Deletion of *retS* (PA4856), *tse3* and tsi3 (PA3484-85) | This study |
| Δ*retS*Δ*tse4_tsi4* | Deletion of *retS* (PA4856), *tse4* and *tsi4* (PA2774-75) | This study |
| Δ*retS*Δ*tse5_tsi5* | Deletion of *retS* (PA4856), *tse5* and *tsi5* (PA2683-84) | This study |
| Δ*retS*Δ*tse6_tsi6* | Deletion of *retS* (PA4856), *tse6* and *tsi6* (PA0092-93) | This study |
| Δ*retS*Δ*tse7_tsi7* | Deletion of *retS* (PA4856), *tse7* and *tsi7* (PA0099-0100) | This study |
| Δ*retS*Δ*tse8_tsi8* | Deletion of *retS* (PA4856), *tse8* and tsi8 (PA4163-64) | This study |
| Δ*rsmA*Δ*rsmN*Δ*tse1_tsi1* | Deletion of *rsmA* (PA0905), *rsmN* (PA5183.1), *tse1* and *tsi1* (PA1844-45) | This study |
| Δ*rsmA*Δ*rsmN*Δ*tse2_tsi2* | Deletion of *rsmA* (PA0905), *rsmN* (PA5183.1), *tse2* and *tsi2* (PA2702-03) | This study |
| Δ*rsmA*Δ*rsmN*Δ*tse3_tsi3* | Deletion of *rsmA* (PA0905), *rsmN* (PA5183.1), *tse3* and tsi3 (PA3484-85) | This study |
| Δ*rsmA*ΔrsmNΔ*tse4_tsi4* | Deletion of *rsmA* (PA0905), *rsmN* (PA5183.1), *tse4* and *tsi4* (PA2774-75) | This study |
| Δ*rsmA*Δ*rsmN*Δ*tse5_tsi5* | Deletion of *rsmA* (PA0905), *rsmN* (PA5183.1), *tse5* and *tsi5* (PA2683-84) | This study |
| Δ*rsmA*Δ*rsmN*Δ*tse6_tsi6* | Deletion of *rsmA* (PA0905), *rsmN* (PA5183.1), *tse6* and *tsi6* (PA1844-45) | This study |
| Δ*rsmA*Δ*rsmN*Δ*tse7_tsi7* | Deletion of *rsmA* (PA0905), *rsmN* (PA5183.1), *tse7* and *tsi7* (PA0099-0100) | This study |
| Δ*rsmA*Δ*rsmN*Δ*tse8_tsi8* | Deletion of *rsmA* (PA0905), *rsmN* (PA5183.1), *tse8* and *tsi8* (PA4163-64) | This study |
| Δ*rsmA*Δ*tle1_tli1ab* | Deletion of *rsmA* (PA0905), *tle1*, *tli1a* and *tli1b* (PA3290-92) | This study |
| Δ*rsmA*Δ*tle3_tli3* | Deletion of *rsmA* (PA0905), *tle3* and *tli3* (PA0259-60) | This study |
| Δ*rsmA*Δ*tle4_tli4* | Deletion of *rsmA* (PA0905), *tle4* and *tli4* (PA1509-10) | This study |
| Δ*rsmA*Δ*pldA_tli5a* | Deletion of *rsmA* (PA0905), *pldA* and *tli5a* (PA3487-88) | This study |
| Δ*rsmA*Δ*pldB_tli5b1-3* | Deletion of *rsmA* (PA0905), *pldB*, *tli5b1*, *tli5b2* and *tli5b3* (PA5086-89) | This study |
| Δ*rsmA*Δ*tseT_tsiT* | Deletion of *rsmA* (PA0905), *tseT* and *tsiT* (PA3907-08) | This study |
| Δ*rsmA*Δ*tseV_tsiV* | Deletion of *rsmA* (PA0905), *tseV* and *tsiV* (PA0821-22) | This study |
| Δ*rsmA*Δ*vgrG2b_vgrG2bi* | Deletion of *rsmA* (PA0905), *vgrG2b* and *vgrG2bi* (PA0261-62) | This study |
| Δ*rsmA*Δ*ampDh3_ampDh3i* | Deletion of *rsmA* (PA0905), *ampDh3* and *ampDh3i* (PA0807-08) | This study |
| Δ*rsmA*Δ*PA5264_PA5265* | Deletion of *rsmA* (PA0905), *PA5264* and *PA5265* | This study |
| Δ*rsmA*Δ*azu* | Deletion of *rsmA* (PA0905) and *azu* (PA4922) | This study |
| Δ*retS*Δ*tle1_tli1ab* | Deletion of *retS* (PA4856), *tle1*, *tli1a* and *tli1b* (PA3290-92) | This study |
| Δ*retS*Δ*pldA_tli5a* | Deletion of *retS* (PA4856), *pldA* and *tli5a* (PA3487-88) | This study |
| Δ*retS*Δ*pldB_tli5b1-3* | Deletion of *retS* (PA4856), *pldB*, *tli5b1*, *tli5b2* and *tlib3* (PA5086-89) | This study |
| Δ*retS*Δ*tseT_tsiT* | Deletion of *retS* (PA4856), *tseT* and *tsiT* (PA3907-08) | This study |
| Δ*retS*Δ*ampDh3_ampDh3i* | Deletion of *retS* (PA4856), *ampDh3* and *ampDh3i* (PA0807-08) | This study |
| Δ*pilA* | Deletion of *pilA* (PA4525) | This study |
| Δ*rsmA*Δ*pilA* | Deletion of *rsmA* (PA0905) and *pilA* (PA4525) | This study |
| Δ*retS*Δ*pilA* | Deletion of *retS* (PA4856) and *pilA* (PA4525) | This study |
| Δ*rsmA*Δ*pilA*Δ*tssB1*Δ*tssB2* Δ*tssB3* | Deletion of *rsmA* (PA0905), *pilA* (PA4525), *tssB1* (PA0083), *tssB2* (PA1657) and *tssB3* (PA2365) | This study |
|  |  |  |
| Δ*rsmA*Δ*pilA*Δ*tseT_tsiT* | Deletion of *rsmA* (PA0905), *pilA* (PA4525), *tseT* and *tsiT* (PA3907-08) | This study |
| Δ*rsmA*Δ*pilA*Δ*tse5_tsi5* | Deletion of *rsmA* (PA0905), *pilA* (PA4525), *tse5* and *tsi5* (PA2683-84) | This study |
