# Supplementary material for: Effectiveness of Pseudomonas aeruginosa type VI secretion system relies on toxin potency and type IV pili-dependent interaction
Source: PLoS Pathog. 2023 May 30;19(5):e1011428. doi: 10.1371/journal.ppat.1011428 (PMC10281587; doi:10.1371/journal.ppat.1011428)
Supplement: S2 Table — (DOCX) [file ppat.1011428.s023.docx]

**S2 Table List of plasmids used in this study**

| **Plasmid constructs** | **Features** | **Source** |
| --- | --- | --- |
| pCR-BluntII-TOPO | Blunt sub-cloning vector, Km^R^ | Invitrogen |
| pKNG101 | Suicide vector, Sm^R^ | Laboratory collection |
| pKNG101_Δ*rsmN* | Suicide vector to delete *rsmN* (*PA5183.1*) from *P. aeruginosa*, Sm^R^ | This study |
| pKNG101_Δ*tssB1* | Suicide vector to delete *tssB1* (*PA0083*) from *P. aeruginosa*, Sm^R^ | Laboratory collection |
| pKNG101_Δ*tssB2* | Suicide vector to delete *tssB2* (*PA1657*) from *P. aeruginosa*, Sm^R^ | Laboratory collection |
| pKNG101_Δ*tssB3* | Suicide vector to delete *tssB3* (*PA2365*) from *P. aeruginosa*, Sm^R^ | Laboratory collection |
| pKNG101_Δ*tse1tsi1* | Suicide vector to delete *tse1* and *tsi1* (PA1844-45) from *P. aeruginosa*, Sm^R^ | Laboratory collection |
| pKNG101_Δ*tse2tsi2* | Suicide vector to delete *tse2* and *tsi2* (PA2702-03) from *P. aeruginosa*, Sm^R^ | Laboratory collection |
| pKNG101_Δ*tse3tsi3* | Suicide vector to delete *tse3* and *tsi3* (PA3484-85) from *P. aeruginosa*, Sm^R^ | Laboratory collection |
| pKNG101_Δ*tse4tsi4* | Suicide vector to delete *tse4* and *tsi4* (PA2774-75) from *P. aeruginosa*, Sm^R^ | Laboratory collection |
| pKNG101_Δ*tse5tsi5* | Suicide vector to delete *tse5* and *tsi5* (PA2683-84) from *P. aeruginosa*, Sm^R^ | This study |
| pKNG101_Δ*tse6tsi6* | Suicide vector to delete *tse6* and *tsi6* (PA0092-93) from *P. aeruginosa*, Sm^R^ | This study |
| pKNG101_Δ*tse7tsi7* | Suicide vector to delete *tse7* and *tsi7* (PA0099-0100) from *P. aeruginosa*, Sm^R^ | This study |
| pKNG101_Δ*tse8tsi8* | Suicide vector to delete *tse8* and *tsi8* (PA4163-64) from *P. aeruginosa*, Sm^R^ | This study |
| pKNG101_Δ*tle1tli1ab* | Suicide vector to delete *tle1*, *tli1a* and *tli1b* (PA3290-92) from *P. aeruginosa*, Sm^R^ | Laboratory collection |
| pKNG101_Δ*tle3tli3* | Suicide vector to delete *tle3* and *tli3* (PA0259-60) from *P. aeruginosa*, Sm^R^ | Laboratory collection |
| pKNG101_Δ*tle4tli4* | Suicide vector to delete *tle4* and *tli4* (PA1509-10) from *P. aeruginosa*, Sm^R^ | Laboratory collection |
| pKNG101_Δ*pldAtli5a* | Suicide vector to delete *pldA* and *tli5a* (PA3487-88) from *P. aeruginosa*, Sm^R^ | Laboratory collection |
| pKNG101_Δ*pldBtli5b1-3* | Suicide vector to delete *pldB*, *tli5b1*, *tli5b2* and *tli5B3* (PA5086-89) from *P. aeruginosa*, Sm^R^ | Laboratory collection |
| pKNG101_Δ*tseTtsiT* | Suicide vector to delete *tseT* and *tsiT* (PA3907-08) from *P. aeruginosa*, Sm^R^ | This study |
| pKNG101_Δ*tseVtsiV* | Suicide vector to delete *tseV* and *tsiV* (PA0821-22) from *P. aeruginosa*, Sm^R^ | This study |
| pKNG101_Δ*vgrG2b_vgrG2bi* | Suicide vector to delete *vgrG2b* and *vgrG2bi* (PA0261-62) from *P. aeruginosa*, Sm^R^ | Laboratory collection |
| pKNG101_Δ*ampDh3_ampDh3i* | Suicide vector to delete *ampDh3* and *ampDh3i* (PA0807-08) from *P. aeruginosa*, Sm^R^ | This study |
| pKNG101_Δ*azu* | Suicide vector to delete *azu* (PA4922) from *P. aeruginosa*, Sm^R^ | This study |
| pKNG101_Δ*pilA* | Suicide vector to delete *pilA* (PA4525) from *P. aeruginosa*, Sm^R^ | This study |
| pKNG101_*tssB1-mScarlet-I* | Suicide vector to deliver TssB1-mScarlet-I fusion into *P. aeruginosa*, Sm^R^ | Laboratory collection |
| pKNG101_*tssB2-sfGFP* | Suicide vector to deliver TssB2-sfGFP fusion into *P. aeruginosa*, Sm^R^ | Laboratory collection |
| pUCP22 | Plasmid containing promoterless GFPmut3b, Gm^R^ | Tim Tolker-Nielsen, University Copenhagen |
| pUCP22_*tssA1*_transcrip. | Plasmid containing GFPmut3b under *tssA1* transcriptional control, Gm^R^ | This study |
| pUCP22_*tssA1*_transl. | Plasmid containing GFPmut3b under *tssA1* translational control, Gm^R^ | This study |
| *pUCP22*_*tssA2*_transcrip. | Plasmid containing GFPmut3b under *tssA2* transcriptional control, Gm^R^ | This study |
| pUCP22_*tssA2*_transl. | Plasmid containing GFPmut3b under *tssA2* translational control, Gm^R^ | This study |
| pUCP22_*tssB3*_transcrip. | Plasmid containing GFPmut3b under *tssB3* transcriptional control, Gm^R^ | This study |
| pUCP22_*tssB3*_transl. | Plasmid containing GFPmut3b under *tssB3* translational control, Gm^R^ | This study |
| miniCTX-pX2-mCherry | mini-CTX suicide vector carrying mCherry under pX2 promoter, Tc^R^ | Knut Drescher Lab, Biozentrum University of Basel |
| miniCTX-pX2-sfGFP | mini-CTX suicide vector carrying sfGFPunder pX2 promoter, Tc^R^ | Knut Drescher Lab, Biozentrum University of Basel |
| pBBRMCS-5 | pBBRMCS-5 empty vector, Gm*R* | Laboratory collection |
| pBBR*tsi2* | pBBRMCS-5 carrying full length *tsi2*, Gm^R^ | This study |
| pBBR*tsi5* | pBBRMCS-5 carrying full length *tsi5*, Gm^R^ | This study |
| pBBR*tsiT* | pBBRMCS-5 carrying full length *tsiT*, Gm^R^ | This study |
| pBBR*tli3-HA* | pBBRMCS-5 carrying 3’HA tagged *tli3*, Gm^R^ | Laboratory collection |
