# Supplementary material for: Effectiveness of Pseudomonas aeruginosa type VI secretion system relies on toxin potency and type IV pili-dependent interaction
Source: PLoS Pathog. 2023 May 30;19(5):e1011428. doi: 10.1371/journal.ppat.1011428 (PMC10281587; doi:10.1371/journal.ppat.1011428)
Supplement: S3 Table — (DOCX) [file ppat.1011428.s024.docx]

**S3 Table List of primers used in this study**

| **Name** | **Sequence^a^** | **Description** |
| --- | --- | --- |
| **Vector primers** | | |
| UpKn | CTCATCAGTGAAATCCAGGG | Primer flanking MCS of pKNG101 |
| RpKn | CATATCACAACGTGCGTGGA | Primer flanking MCS of pKNG101 |
| M13 Fw | TGTAAAACGACGGCCAGT | M13 forward sequencing primer |
| M13 Rw | CAGGAAACAGCTATGACC | M13 reverse sequencing primer |
| OAL1604 | CGCGCGTAATACGACTCACT | Primer flanking MCS of mini-CTX plasmids |
| OAL1605 | CCGTCCTTGCTGAATTAGCTT | Primer flanking MCS of mini-CTX plasmids |
| **Construction of mutator plasmids and screening of gene deletions** | | |
| OAL3239 | ATGAT**GGGCCC**GCTCCAGGTTGAGCTGATTGAGGC | Constructing mutator for *rsmN* deletion P1 (*Sma*I) |
| OAL3240 | CAACTCGTCGAAACCCATGTTCCGCGT | Constructing mutator for *rsmN* deletion P2 |
| OAL3241 | ATGGGTTTCGACGAGTTGAAGACGGCACCG | Constructing mutator for *rsmN* deletion P3 |
| OAL3242 | ATCAT**GGATCC**TAATCGCGTTCGGCCTGCTG | Constructing mutator for rsmN deletion P1 (*Bam*HI) |
| OAL6642 | GCTTGCCGAAGCTGATGTGT | Screening *rsmN* deletion |
| OAL6643 | CTCGACGCTGGAGCAATGTT | Screening *rsmN* deletion |
| OAL2661 | CGACCCCACCTTCCGTATCAAC | Screening *tssB1* deletion / fluorescent fusion |
| OAL2662 | CGATGTAGCGGGAGTCCTCG | Screening *tssB1* deletion / fluorescent fusion |
| OAL1594 | CAGGCGATGCGGGAAGTCGAAA | Screening *tssB2* deletion / fluorescent fusion |
| OAL1595 | TCTGCCACTTGGCGAACTGC | Screening *tssB2* deletion / fluorescent fusion |
| OAL3149 | GAAGGACTCCGACTCGATGAAC | Screening *tssB3* deletion |
| OAL3150 | GACGTTCGACAGCTTCTCCA | Screening *tssB3* deletion |
| OAL1295 | CACTTGGCGGTAAAGATCG | Screening *tse1tsi1* deletion |
| OAL1296 | GATGGCCTGGATCACGTC | Screening *tse1tsi1* deletion |
| OAL1283 | GCTGCTGCTCGGCCTGTTC | Screening *tse2tsi2* deletion |
| OAL1284 | AGGCGCCTGAACTGTTCGT | Screening *tse2tsi2* deletion |
| OAL1345 | CCATTACGCCGAACTCACC | Screening *tse3tsi3* deletion |
| OAL4465 | CGGGGTGAAGGCGAGGAAGG | Screening *tse3tsi3* deletion |
| OAL4649 | CCGACGCGGCGTAATAGG | Screening *tse4tsi4* deletion |
| OAL4650 | CTGGATGCCCGGCCAGGC | Screening *tse4tsi4* deletion |
| OAL6254 | ATTATA**GTCGAC**CACGGGTCGCCTCGATCTTCACC | Constructing mutator for *tse5tsi5* deletion P1 (*Sal*I) |
| OAL6255 | ATGAGCGGCGACGAGTGAGGCCGACCC | Constructing mutator for *tse5tsi5* deletion P2 |
| OAL6256 | TCACTCGTCGCCGCTCATCTATCCATCCTTCCTTG | Constructing mutator for *tse5tsi5* deletion P3 |
| OAL6257 | ATTATA**GGGCCC**GCAACGAGACGGTCAGGATCGGC | Constructing mutator for *tse5tsi5* deletion P4 (*Apa*I) |
| OAL6258 | CAGCACGTCGGGGTGATC | Screening *tse5tsi5* deletion |
| OAL6259 | ACGAGACGGTGCATGTGAAG | Screening *tse5tsi5* deletion |
| OAL6260 | ATTATA**GGATCC**CAACAAGAGCATCGGCCACGAC | Constructing mutator for *tse6tsi6* deletion P1 (*Bam*HI) |
| OAL6261 | ATGGATGCGCTGCCCTGAGCGCAACACC | Constructing mutator for *tse6tsi6* deletion P2 |
| OAL6262 | TCAGGGCAGCGCATCCATGCGTCGGTAC | Constructing mutator for *tse6tsi6* deletion P3 |
| OAL6263 | ATTATA**GGGCCC**CAGGAAAGCCTTGATTCGCGAGC | Constructing mutator for *tse6tsi6* deletion P4 (*Apa*I) |
| OAL3352 | AGTTATACATCCACGCCGAGC | Screening *tse6tsi6* deletion |
| OAL3353 | GAAAGGGGAGATGCGTGACA | Screening *tse6tsi6* deletion |
| OAL6264 | ATTATA**GGATCC**CAACGGCTTCATTCCCGGCG | Constructing mutator for *tse7tsi7* deletion P1 (*Bam*HI) |
| OAL6265 | TGCTGGCCGTTGGCCATCAGGCAGCC | Constructing mutator for *tse7tsi7* deletion P2 |
| OAL6266 | ATGGCCAACGGCCAGCAATGGACGCG | Constructing mutator for *tse7tsi7* deletion P3 |
| OAL6267 | ATTATA**GGGCCC**GCCAAGGGCGGACAGCAG | Constructing mutator for *tse7tsi7* deletion P4 (*Apa*I) |
| OAL3307 | TGGAAGGCGAGCTGGGAC | Screening *tse7tsi7* deletion |
| OAL3308 | TGCCAGGCGAGCAGCA | Screening *tse7tsi7* deletion |
| OAL6268 | ATTATA**GGATCC**CGAGAGCAATCCGCGCAGC | Constructing mutator for *tse8tsi8* deletion P1 (*Bam*HI) |
| OAL6269 | TCAGTCGCGCTCGATCATGCTGTCACCGCC | Constructing mutator for *tse8tsi8* deletion P2 |
| OAL6270 | ATGATCGAGCGCGACTGAGCGCTTGCC | Constructing mutator for *tse8tsi8* deletion P3 |
| OAL6271 | ATTATA**GGGCCC**CGCATCGGCTACATGGTCCTG | Constructing mutator for *tse8tsi8* deletion P4 (*Apa*I) |
| OAL6272 | CTGCACAGGTTGACGATGC | Screening *tse8tsi8* deletion |
| OAL6273 | CGACAGCGAGTTCCACTACG | Screening *tse8tsi8* deletion |
| OAL4321 | GCGCTGTTCAAATTGCTGGAG | Screening *tle1tli1ab* deletion |
| OAL4322 | AGAACAGGCCAGGCATTCTAG | Screening *tle1tli1ab* deletion |
| OAL3293 | CCGGGAAAGACGTTGAAGGA | Screening *tle3tli3* deletion |
| OAL3294 | GTAGGTTCGGATGGCGGTAG | Screening *tle3tli3* deletion |
| OAL2586 | GTTTTCAGCGACCCCTACCTC | Screening *tle4tli4* deletion |
| OAL2053 | CCGCAGCAAACCCTCCAG | Screening *tle4tli4* deletion |
| OAL3231 | CCGGGCAGAAGATGGTGATC | Screening *pldAtli5a* deletion |
| OAL3232 | AGGACGATGCAATTGGTGGT | Screening *pldAtli5a* deletion |
| OAL3237 | GAACTGGCCACCTTGCATTC | Screening *pldBtli5b1-3* deletion |
| OAL3238 | GGGCGACGAGATCCATTTCA | Screening *pldBtli5b1-3* deletion |
| OAL6274 | ATTATA**GTCGAC**GCGCTGGAACAGGTCATGACC | Constructing mutator for tseTtsiT deletion P1 (*Sal*I) |
| OAL6275 | TCAGCCGCGGCCGCTCATGCCGGTCTC | Constructing mutator for *tseTtsiT* deletion P2 |
| OAL6276 | ATGAGCGGCCGCGGCTGACGACGTACG | Constructing mutator for *tseTtsiT* deletion P3 |
| OAL6277 | ATTATA**GGGCCC**GGCCTGTAGGGCGAATAACCG | Constructing mutator for *tseTtsiT* deletion P4 (*Apa*I) |
| OAL6278 | CATGGCCTCCTGGCTGTG | Screening *tseTtsiT* deletion |
| OAL6279 | GCCGGGTGGCGATGAAG | Screening *tseTtsiT* deletion |
| OAL6548 | ATTATA**GGATCC**CGAGTCTCGCACCGAGG | Constructing mutator for *tseVtsiV* deletion P1 (*Bam*HI) |
| OAL6549 | ATGACCAAGCGCCTGTAGGGGCTTACG | Constructing mutator for *tseVtsiV* deletion P2 |
| OAL6550 | CTACAGGCGCTTGGTCATGTCGTAGCTGACC | Constructing mutator for *tseVtsiV* deletion P3 |
| OAL6551 | ATTATA**ACTAGT**CCTTAACCGCAACAGCGTG | Constructing mutator for *tseVtsiV* deletion P4 (*Spe*I) |
| OAL6552 | TCACCTTTGATAGCATCCTGGC | Screening *tseVtsiV* deletion |
| OAL6553 | GTGACGCTGGGTAGTATCGG | Screening *tseVtsiV* deletion |
| OAL3320 | TGAACTAGTGTAACGCTTGCGGATGATCTTG | Screening *vgrG2bvgrG2bi* deletion |
| OAL3321 | CCCGACGACATTGATGGTGT | Screening *vgrG2bvgrG2bi* deletion |
| OAL6824 | ATTATAGGATCCAGTAGCCGCTCTGTCGAGGGT | Constructing mutator for *ampDh3ampDh3i* deletion P1 |
| OAL6825 | TCAGCCATCGAGACCGCGATAGCTGTTGTAGTCG  ATGGTCAGCATGG | Constructing mutator for *ampDh3ampDh3i* deletion P2 |
| OAL6826 | GACTACAACAGCTATCGCGGTCTCGATGGCTGAG  CATTG | Constructing mutator for *ampDh3ampDh3i* deletion P3 |
| OAL6827 | ATTATAACTAGTAACGCGGCGATCCTGATCGTC | Constructing mutator for *ampDh3ampDh3i* deletion P4 |
| OAL6671 | CCGCTGGCACGACGCTAG | Screening *ampDh3ampDh3i* deletion |
| OAL6672 | ACCTGGCGATCGGCATCGT | Screening *ampDh3ampDh3i* deletion |
| OAL6673 | ATTATA**ACTAGT**TGGCCTACAACAAGGACAACCAGG | Constructing mutator for *PA5264PA5265* deletion P1 (*Spe*I) |
| OAL6674 | AGCGGATTCGAGCACTGATGCTGAGTTATTCCGC | Constructing mutator for *PA5264PA5265* deletion P2 |
| OAL6675 | TCAGTGCTCGAATCCGCTCATGCCTCGCTC | Constructing mutator for *PA5264PA5265* deletion P3 |
| OAL6676 | ATTATA**GGATCC**ACATCGAGCACGACCAGAAGATCC | Constructing mutator for *PA5264PA5265* deletion P4 (*Bam*HI) |
| OAL6677 | TCATCGACCTGCCCGACC | Screening *PA5264PA5265* deletion |
| OAL6678 | AAGACCCGCAGCGTCTTCA | Screening *PA5264PA5265* deletion |
| OAL6408 | ATTATA**ATGCAT**CCTGGAACAACTGGCCGGC | Constructing mutator for *azu* deletion P1 (*Nsi*I) |
| OAL6409 | ATGCTACGTCTGAAGTGATGCGCGAGCG | Constructing mutator for *azu* deletion P2 |
| OAL6410 | TCACTTCAGACGTAGCATGGAGCAGCCTC | Constructing mutator for azu deletion P3 |
| OAL6411 | ATTATA**GGATCC**CGATCTCGGCCTCCTGCAGG | Constructing mutator for *azu* deletion P4 (*BamH*I) |
| OAL6412 | AGCTGTATCCCTGCGAAGG | Screening *azu* deletion |
| OAL6413 | GGCAAGGGCCAGAAGATCG | Screening *azu* deletion |
| OAL6508 | ATTATA**GGATCC**CGCATAGCACCCGGCAAG | Constructing mutator for *pilA* deletion P1 (*Bam*HI) |
| OAL6509 | ATGAAAGCTGATAACTAAGGTGATCGAAGGTG | Constructing mutator for *pilA* deletion P2 |
| OAL6510 | CTTAGTTATCAGCTTTCATGAATCTCTCCGT | Constructing mutator for *pilA* deletion P3 |
| OAL6511 | ATTATA**GGTACC**GTGTTGGCGGACCAGCT | Constructing mutator for *pilA* deletion P4 (*Kpn*I) |
| OAL6512 | GCCACAACCATCGCATCGG | Screening *pilA* deletion |
| OAL6513 | CGACCAGAATCGCTTCGGTC | Screening *pilA* deletion |
| **Construction of reporter plasmids** | | |
| OAL5919 | TAATAAGGTACCGGTCAGCTGTCCCTGGTCCAGT | Constructing transcriptional / translational fusion for *tssA1* promoter (*Kpn*I) |
| OAL6365 | ATTATA**GGATCC**GGCAGCCAGCAAAACGGGTAC | Constructing transcriptional fusion for *tssA1* promoter (*Bam*HI) |
| OAL5921 | TAATAA**GGTACC**GCGCAGTAGTGTTCAGGCCAGT | Constructing transcriptional / translational fusion for *tssA2* promoter (*Kpn*I) |
| OAL6366 | ATTATAGGATCCATGCGAGGAGAGCTTGCTCGAAT | Constructing transcriptional fusion for *tssA2* promoter (*Bam*HI) |
| OAL5923 | TAATAA**GGTACC**CTCCATACCGCGAACTGCTGCC | Constructing transcriptional / translational fusion for *tssB3* promoter (*Kpn*I) |
| OAL6367 | ATTATA**GGATCC**GTCCAGCTTGTGCTGCGTACTC | Constructing transcriptional fusion for *tssB3* promoter (*Bam*HI) |
| OAL6530 | TTTACGCATGGCAGCCAGCAAAACGG | Constructing translational fusion for *tssA1* promoter P2 |
| OAL6531 | CTGGCTGCCATGCGTAAAGGAGAAGAACTTTTCAC | Constructing translational fusion for *tssA1* promoter P3 |
| OAL6532 | GAGTCAGCTCAGCTAATAAGCTTATTTGTAT | Constructing translational fusion for *tssA1* promoter P4 |
| OAL6533 | ATTATA**GCATGC**AATGCGAGGAGAGCTTGCTC | Constructing translational fusion for *tssA2* promoter (*Sph*I) |
| OAL6534 | ATTATA**GCATGC**GGTCCAGCTTGTGCTGCG | Constructing translational fusion for *tssB3* promoter (*Sph*I) |
| OAL5919 | TAATAA**GGTACC**GGTCAGCTGTCCCTGGTCCAGT | Constructing transcriptional / translational fusion for *tssA1* promoter (*Kpn*I) |
| Tsi2-SD-For | CCGGAATTCGAAGGAGGTGAACCATGAACCTGAAACCC | Constructing complementing clone of *tsi2* in pBBRMCS-5 |
| Tsi2Rev | TGCTCTAGATCAGGATGCCGGCTCTTCGATC | Constructing complementing clone of *tsi2* in pBBRMCS-5 |
| Tsi5-SD-For | CCGGAATTCGAAGGAGTCGTCAAATGCCCACTGAAGG | Constructing complementing clone of *tsi5* in pBBRMCS-5 |
| Tsi5Rev | TGCTCTAGATCACTCGTCCAGGTAGCCGTCC | Constructing complementing clone of *tsi5* in pBBRMCS-5 |
| TsiT-SD-For | CCGGAATTCGAAGGAGATTGCGCATGACGGACGCCAAG | Constructing complementing clone of *tsiT* in pBBRMCS-5 |
| TsiTRev | TGCTCTAGATCAGCCGCGCAGCGGCG | Constructing complementing clone of *tsiT* in pBBRMCS-5 |

^a^Restriction sites are shown in bold when mentioned in the description
