# Supplementary material for: Effectiveness of Pseudomonas aeruginosa type VI secretion system relies on toxin potency and type IV pili-dependent interaction
Source: PLoS Pathog. 2023 May 30;19(5):e1011428. doi: 10.1371/journal.ppat.1011428 (PMC10281587; doi:10.1371/journal.ppat.1011428)
Supplement: S4 Table — (DOCX) [file ppat.1011428.s025.docx]

**S4 Table Simulation parameters**

| **Parameter** | **Value(s)** | **Source** |
| --- | --- | --- |
| **Simulation initiation** | | |
| Inoculum radius* | 110μm |  |
| Inoculum density (OD)** | 0.1, 8.0, 4.0, 2.0, 1.0, 0.5, 0.25, 0.125 |  |
| Attacker to prey ratio in inoculum | 0.5, 0.45, 0.4, 0.35, 0.3, 0.25, 0.2, 0.15, 0.1, 0.05 |  |
| **Biophysics** | | |
| Time step | 0.025h | [1] |
| Cell growth drag | 10 | [1] |
| Max contacts | 24 | [1] |
| Number of sub-steps | 8 | [1] |
| Division orientation noise | 0.10% | [1] |
| **Cells parameters** | | |
| Cell radius | 0.5μm | Estimated from single cell microscopy images |
| Target division length | 3μm | Estimated from single cell microscopy images |
| Cell division noise | 0.5 | [2] |
| Maximum growth rate | 1 h^-1^ | [2] |
| **T6SS parameters** | | |
| T6SS firing rate | 1, 5, 25, 50, 100, 200 | [3] |
| Toxin lethal dose | 1, 5, 10, 20, 50 | [3] |
| Toxin lysis delay (min) | 3, 60, 120, >simulation time | [3] |
| Fraction of attacker population actively firing | 1, 0.5, 0.25, 0.125, 0.0625 | This study |
| Upfront T6SS cost | 0 | [4] |
| Cost per T6SS firing event | 0.00001 | [4] |
| Needle length | 0.5μm | [4] |
| Minimum needle penetration | 10nm | [3] |

* 1μL spot drying on agar will result in inoculum area of 7.55 * 10^6^ μm^2^ (Estimated from whole colony microscopy images)

** 2.04*10^5^ colony forming units per 1μL of OD600=1.0 culture as per Kim et.al. 2012

References

1. Rudge TJ, Steiner PJ, Phillips A, Haseloff J. Computational modeling of synthetic microbial biofilms. ACS Synth Biol. 2012;1(8):345-52. Epub 20120810. doi: 10.1021/sb300031n. PubMed PMID: 23651288.
2. Bottery MJ, Passaris I, Dytham C, Wood AJ, van der Woude MW. Spatial Organization of Expanding Bacterial Colonies Is Affected by Contact-Dependent Growth Inhibition. Curr Biol. 2019;29(21):3622-34 e5. Epub 20191017. doi: 10.1016/j.cub.2019.08.074. PubMed PMID: 31630946; PubMed Central PMCID: PMCPMC6839403.
3. Smith WPJ, Vettiger A, Winter J, Ryser T, Comstock LE, Basler M, et al. The evolution of the type VI secretion system as a disintegration weapon. PLoS Biol. 2020;18(5):e3000720. Epub 20200526. doi: 10.1371/journal.pbio.3000720. PubMed PMID: 32453732; PubMed Central PMCID: PMCPMC7274471.
4. Smith WPJ, Brodmann M, Unterweger D, Davit Y, Comstock LE, Basler M, et al. The evolution of tit-for-tat in bacteria via the type VI secretion system. Nat Commun. 2020;11(1):5395. Epub 20201026. doi: 10.1038/s41467-020-19017-z. PubMed PMID: 33106492; PubMed Central PMCID: PMCPMC7589516.
