# Supplementary material for: Effectiveness of Pseudomonas aeruginosa type VI secretion system relies on toxin potency and type IV pili-dependent interaction
Source: PLoS Pathog. 2023 May 30;19(5):e1011428. doi: 10.1371/journal.ppat.1011428 (PMC10281587; doi:10.1371/journal.ppat.1011428)
Supplement: Attachment — Submitted filename: RESPONSE TO REVIEWERS.docx [file ppat.1011428.s026.docx]

**POINT BY POINT ANSWER TO REVIEWER’S COMMENTS**

We are very grateful to the editors and reviewers for providing constructive comments and for giving us the opportunity to improve our manuscript. We have now addressed all the comments and revised the manuscript accordingly. We have paid particular attention to the 3 main points, which were to; i) provide more quantification information for the description of our microscopy images; ii) complement the sensitized mutants by bringing in trans the cognate T6SS immunity and iii) assess the T6SS activity of the *pilA* mutant as compared to the parental strain. All changes made are described here below , under each reviewer comments which are highlighted in blue. Note that additional experiments on immunity mutants complementation and T6SS activity in the *pilA* mutant, which are now included in the revised manuscript, have been conducted by Sujatha Subramoni who has been included as a co-author.

**Reviewers' comments:**

**Part I – Summary**

*Please use this section to discuss strengths/weaknesses of study, novelty/significance, general execution and scholarship.*

***Reviewer #1:*** *In this article, authors perform a systematic deletion of all known pairs of antibacterial T6SS effectors and cognate immunity proteins (E/I) in Pseudomonas aeruginosa. The contribution of individual effectors was analyzed via intraspecies competition between mutants and wild-type cells by imaging mixed bacterial macrocolonies. It was observed that the susceptibility of preys cells varies according to the effector/immunity pair they lack. Also, the initial composition of the community influences the outcome of the competition, with the frequency of contacts between attacker and prey cells at an initial stage being key to the outcome. All the key concepts touched in the articles are already established in the T6SS field. The differential of the study is that it included all known P. aeruginosa T6SS effector/immunity pairs described to date. The article mainly expands on the concept described by LaCourse et al Nat Microbiol 2018, which described the difference in effectiveness of individual effectors and their synergistic mode of action.*

This this is a fair comment and we agree that our study is not the first one to address the concept of “why so many toxins”, while we duly quote the work by la Course and collaborators. Yet we feel that, beyond being comprehensive and systematic, our data clearly demonstrate some of the synergy such as between Tse1 and Tse3, acting concomitantly on peptidoglycan degradation. Such synergy in combining cleavage of glycan chains and peptide bonds simultaneously has not been shown before. Our study also provides a great deal of information in the variation of T6SS toxin potency, and this is clearly of great added value when it comes to the exploitation of knowledge and the design of protective T6SS strains combatting undesired pathogens. Our study also provides rather new concepts, such as the role of type IV pili in bacterial competition, in which the associated “run-away” strategy has a more prominent role in protection as compared to the “avoid stable contact” tactic.

*The methodology chosen to analyze the outcome of the competition (imaging macrocolonies) could have been better explored if authors included some sort of quantification (center and edge of colonies, etc).*

We fully agree with the reviewers, that even though differences are sometime visually striking, quantification will provide a more rigorous interpretation of the data presented. One approach we have thus used to address this comment is shown in the new supplementary figures S8 and S9 Figs, which includes a set of 3 biological replicate macrocolony communities (H1 toxins, *rsmA* background) and analysis of fluorescence signal spatial distribution. Note that because we added several supplementary figures the numbering has now changed as compared to the original version.

Looking at relative fluorescence signal mean intensity as a function of distance from the colony centre, we see that in the given macrocolonies, bacteria appear equally distributed across the whole of the inoculum region independently of the attacker populations T6SS activity or toxin used in the competition. A stark drop in mean prey signal intensity can be seen in competition reliant on delivery of toxins Tse2, Tse4, Tse5, Tse6, and Tse7. Some of this description has now been added in the manuscript on lines 182-202. The mean signal intensity does not necessarily reflect differences in the impact of individual toxins, but the added measure of signal variance is increased in cases where a macrocolony contains multiple prey bacteria segments. When this is converted to a spatial analysis of relative prey bacteria abundance, we see that the trend remains consistent across 3 biological replicate colonies analysed. Where competition effectiveness is visually apparent, spatial analysis helps to confirm the impact. If the competition outcomes are less striking, image analysis here remains unable to provide a fully conclusive answer, as the impact of individual toxins is best evaluated in a broader context across strains delivering toxins at different rates (Fig. 4B). One of the challenges that remains with more quantitative evaluation of the exact extent of toxin impact is due to these communities being inherently highly varied, considering this, a large number of replicate communities would be required. This might be incompatible with the study's aims of evaluating a large number of different toxins using this methodology.

*As the work stands, the outcome if mainly visual and does not bring new insights. The study would have more significance if authors used their E/I mutant strains as attackers in competition with relevant species that are common competitiors of Pseudomonas in natural settings.*

This is also a very reasonable comment. However, the goal of the study was to evaluate the potency of individual T6SS toxin, which we realize can only be observed and compare when using isogenic strain in the attacker/prey competition. Using other strains in the competition, including common P. aeruginosa competitors, will not be possible since these strains would be sensitive to most of the T6SS toxins, which means that the attacker should be engineered to deliver only one toxin at a time, or the prey provided with all the immunities except one. Furthermore the T6SS is only one of the device involved in competition. Using non-isogenic strain will then involve other competitive factors, such as CDI or diffusible toxins, and this could mask some of the T6SS action. In that case the competition would then become strain specific and not T6SS toxin-specific.

*There are several key points in experimental settings that could be responsible for the outcomes and were not discussed/considered by the authors.*

These points are addressed in the reviewer 1 major and minor issues further below.

***Reviewer #2:*** *In the manuscript, the authors describe a study on the role of the P. aeruginosa type VI secretion system (T6SS) in interbacterial competition, when attackers (T6SS+) and prey cells interact on agar surface. The P. aeruginosa chromosome carries genes encoding for 3 distinct T6SSs and diverse killing effectors. Using a comprehensive collection of mutants with calibrated expression/delivery of toxins specific for each T6SS, the authors utilized advanced microscopy methods to image bacterial survival and growth in colonies containing attacker/prey pairs of wild type and mutant bacteria. They also show that the community of structure was strongly influenced by the type of toxin, their quantity, or combination delivered to the prey. Finally, the authors concluded the study by demonstrating a role for twitching motility, mediated by type 4 pili, for the prey to escape attackers.

This is an interesting paper with a large amount of data for explaining a complex community behavior of P. aeruginosa, addressing the role of multiple toxin secreted by the T6SS and bacterial motility (although curiously, flagellar motility was not considered).*

We are grateful to the reviewer for this very clear overview of our work

**Part II – Major Issues: Key Experiments Required for Acceptance**

*Please use this section to detail the key new experiments or modifications of existing experiments that should be absolutely required to validate study conclusions.*

*Generally, there should be no more than 3 such required experiments or major modifications for a "Major Revision" recommendation. If more than 3 experiments are necessary to validate the study conclusions, then you are encouraged to recommend "Reject".*

As indicated previously, we have now addressed the 3 main points, which were to; i) provide more quantification information for the description of our microscopy images; ii) complement the sensitized mutants by bringing in trans the cognate T6SS immunity and iii) assess the T6SS activity of the *pilA* mutant as compared to the parental strain.

*Reviewer #1:*

*1) Use/develop some quantitative method to quantify and analyze the outcomes of the mixed macrocolonies (center and edge).*

We have now addressed this comment as described in the section above.

*2) Complement all E/I mutants with a plasmid containing the immunity protein in order to restore the resistant phenotype.*

As indicated the strains used in competition are isogenic and differ only by the absence of either one of the 23 T6SS immunity genes. We fully agree that it is important to confirm that most of the impact seen in the competition assay is due to the lack of immunity. Out of the 23 sensitized strains, and in order to perform competition assays, we thus selected two strains sensitized against distinct H1-T6SS toxins and two strains sensitized against H2-T6SS toxins. The selected strains were sensitive against Tse2, Tse5, Tle3 or TseT. The cognate immunity genes were cloned into pBBRMCS-5 and introduced in trans in appropriate mutants. These data are now shown in the new supplementary S11 Fig and discussed on lines 203-214. The methods and strains/plasmids Tables have also been amended accordingly, including for the whole colony imaging since it was performed in a different laboratory. As for Tle3 and TseT sensitized strains it is clear that complementation restores growth of the prey strains as seen by the bacterial expansion shown with the red channel. In case of Tse2 this is also clear that expansion of the prey is largely restored (growth zone is 3x larger as compared to the immunity sensitive context). Only for Tse5 the effect is barely visual. Yet, the quantification for *tsi5* complementation has been done using Image J (averaging 3 replicates) and we have plotted integrated density for SFGFP and mCherry in the mix. This is shown in the graph below suggesting a statistically significant difference in the mCherry intensity in the presence of pBBR*tsi5*, whereas SfGFP (attacker) does not show much difference. The integrated density refers to the sum of pixel values divided by the total area of the specific red or green fluorescence. This mild complementation could be due to several factors, such as the low copy number of immunity proteins as compared to the toxins and/or the high toxicity of Tse5. It could be also be due to the fast folding kinetic and insertion of the Tse5 pore in the membrane, when toxin and immunity genes are not co-translated. Complementation of all 23 mutants would be tedious but our new data demonstrate that the experimental procedure we used is fully adequate to unveil T6SS toxins impact.

*3) The fact that their prey E/I mutants are in a different background (repressed T6SS) compared to the attacker WT cells (active T6SS) worries me about the outcomes because the expression of all E/I pairs is probably downregulated in all prey cells. Authors should monitor the expression of all E/I pairs in prey cells and/or perform all the competitions in the same active T6SS background.*

We apologize if this was confusing, but in all experiments conducted in our paper, T6SS toxin mediated competition is assessed in competition between T6SS toxin/immunity mutant and a parental strain of the same regulatory background. So in essence, and as requested by the reviewer competition occurs in the same active T6SS background. The competition is near isogenic – since the only differences between toxin/immunity sensitive strain (tagged with mCherry) and parental competitor (sfGFP) are the presence/absence of specific toxin and a fluorescent tag. Parental strains with inactive T6SSs contain 3 additional mutation in *tssB1/B2/B3* genes.

This means that although T6SS associated genes expression levels would differ between competition within distinct *gac-rsm* mutational backgrounds, these would still remain matched between any 2 competitors within a mixed macrocolony.

Where possible (Fig 3, Fig4A, FigS7, FigS8 (now FigS10), and FigS9 (now FigS12)), figures have been amended to more explicitly show the genetic background of each of the competitors, rather than just stating “T6SS+” or “T6SS–“. We hope that this will aid with interpretation of this data.

*On this topic, the quantification of secreted individual effectors in attacker cells using western blot is also important as authors claim in the text that the difference between retS, rsmA and rsmA/N mutants is due to the amount of effectors injected, which in my opinion seems to be due to the number of firing events.*

We agree with the reviewer that this will be an ideal approach. However the level of T6SS effectors secreted in vitro is generally very low and barely detectable, and we have no antibodies available to detect with confidence T6SS toxins and effectors. The best readout we generally used for assessing secretion is Hcp release which we have shown here in Figure 1.

*4) Authors should discuss and consider that the outcome of the killing and their results is due to the capacity of the prey cells of repair the damage caused by individual toxins, which is directly associated with the growth phase of the individual prey cells.*

Although this is an interesting point raised by the reviewer, we are not aware that published work reports any evidence about individual cells growth phase having impact on T6SS activity. The number of individual interacting bacterial pairs within a community is very high. the overall impact is determined by the overall average outcome. This means that overall average killing capacity of attacker population as a whole and damage repair capabilities of prey population as a whole is what determines the overall outcome. At a scale of whole population, it is thus impossible to meaningfully detangle the impact of individual cells growth phase.

*5) The initial cell density (OD600nm) and ratio between attacker and prey (e.g. 1:2) should be clear in all experiments/figures.*

Figure descriptions have been amended to include this information.

*6) Fig 6F needs a control to measure T6SS expression and secretion levels and firing frequency in pilA mutant*

We have now considered the secretion activity in the *pilA* mutant as compared to the corresponding isogenic parental strain. We used western blot to monitor production and secretion of either Hcp1 and Hcp2. There are no noticeable differences in T6SS activity when comparing the *rsmA* and *rsmA/pilA* strains and this is now shown in a new supplementary figure S18 Fig, and briefly commented in the main text on lines 353-355. We also performed western blot for the production of Hcp3 and here also did not notice any significant differences (S18 Fig, panel C). There is no reliable secretion assay for Hcp3 so this is not shown. As for the H3-T6SS we thus also performed further analysis on expression level using transcriptional and translational reporter fusion to the *tssB3* gene, and again did not notice any differences in T6SS activity when comparing *rsmA* and *rsmA/pilA* mutant. These data have not been included in the paper but shown here in the figure below where it is clear that elevated activity of H3-T6SS is observable in the *rsmA/pilA* (blue) and *rsmA* (green) strains as compared to WT (black) or *pilA* (red) strains. Note that since this series of western blots have been done in different laboratories the methods have been amended to reflect both set of experiments, that is those shown in Fig 1 and S2 Fig., and those shown in the new S18 Fig.

*Reviewer #2: None*

***Part III – Minor Issues: Editorial and Data Presentation Modifications***

*Please use this section for editorial suggestions as well as relatively minor modifications of existing data that would enhance clarity.*

***Reviewer #1:*** *1) Fig 1B and C: Change the colours and include patterns to facilitate visualization.*

The colours we used in the graphs are compatible with colour universal design. We also feel that due to the high number of data points, inclusion of patterns would obscure error bars and make it difficult to distinguish curves with close values. An illustrative example of graphs with and without patterns is included below of reviewer perusal.

*Fig. 1D and E: correct the name of the mutant strain tssB.*

The "t" from "*tssB*" was partially cropped from panel E, and the strain names in the figure have now been corrected.

*Fig 1F: Include scale bar and phase contrast images in another row or merged.*

The brightfield images have now been included and the scale bar of 2μm added below.

*2) S3Fig: the difference in temperatures is not clear. Label the figures to facilitate.*

All measurements in this figure are performed at the same temperature which is 25°C

*3) Videos: Include scale bars and time stamps. Labels with strain names will make it easier too!*

The error bars and time stamps have been added to videos.

*4) Several supplementary figures (e.g. S5, S9 (now S12), S10 (now S13), S11 (now S14), etc) are low resolution or labeled with a different figure number (probably from a previous submission that was not reformatted properly). Please correct those.*

Images in the amended figure set are of higher quality and numbering is now only in the file names. We could possibly provide even higher resolution figures, but the limitation is imposed by the submission guidelines.

*5) Names of mutant strains should be properly written with a delta letter from greek alphabet before the gene name. The way it is written is the text is very confusing and informal (e.g. "retS background").*

Name of mutant strains have been reviewed all through and a Δ has been added where appropriate.

*6) Table S1: gene names should be in italic.*

All gene names in Table S1 are now with italics.

*7) Fig 3: there is not A and B as described in the text.*

The panels in the figure have now been changed and compare the impact of individual T6SS toxins in a Δ*rsmA* and Δ*retS* background. The panels now also contain A and B lettering.

*8) Fig S7 and S8 seem to be misplaced.*

The citation order for Fig S7 and S8 has now been checked and corrected. Also note that Fig S7 has been changed and now includes WT and *rsmA/rsmN* instead of *retS* and *rsmA/rsmN*, which is to reflect the fact that the new Fig 3 is now showing *rsmA* and *retS* instead of WT and *rsmA*. Note also that because several additional supplementary figures have been included Fig S8 is now Fig S10.

*9) Standardize Gac/Rsm or Gac-Rsm*

This has now been changed systematically to Gac/Rsm

*10) Line 423: through instead of trough*

This typo was in fact found at several other places as well and has been corrected all through.

***Reviewer #2:***

*1. The construction of plasmid reporter systems used in SFig 1 is confusing, perhaps because it was described inadequately. It took some time for this reviewer to understand (possibly incorrectly) based on the rather brief description in the Materials and Methods that each construct used a similar the PCR amplicon (+30 to -500) relative to the start codon of tssA genes. These were than fused to the promoterless GFPmut3b  gene. Presumably, one set was fused to the coding sequence of GFP, another set created transcriptional fusions. This should be explained clearly, with appropriate detail, without a reader’s need to consult the primer list in the S3Table. The list of primers also lacks a Legend, making the reader guess about a few features (for example, where is the fusion junction for translational fusions and what is the meaning of (restriction sites) in several descriptions.*

The description in the methods has been modified to get more clarity and now includes the following on lines 555-559:

“Translational reporters were designed such that 10 leading amino acids encoded from the *tss* gene were translationally fused to *gfp*, such that fluorophore translation occurs from the same RBS as the *tss* genes. For transcriptional reporters, the *tss* promoter is used and an additional RBS has been included to allow for GFP production independently of translation of the given *tss* gene.”

A figure is also shown here below to differentiate the design of the transcriptional vs translational fusions.

Note that in the S3 Table the restriction sites indicated in the description have been highlighted in bold letters in the sequence.

*Moreover, one can quibble about the terminology “transcriptional/translational” fusion, since translation depends on transcription, and these plasmids did not address the extent of translational regulation at all. Yet the authors claim they can differentiate this, as indicated in the description and interpretation of Figure 2 and S3Figures and lines 119-120).*

Just to clarify, in the transcriptional reporters, theT6SS gene promoter region is followed by an extra RBS. This means that Gfp production is mostly independent of the post transcriptional control by RsmA/N. Indeed, even if these translational repressors bind to the RBS upstream of the *tss* gene, it should not hamper translation of Gfp. Instead, in the translational reporters, Gfp production will be subjected to the control by RsmA/N.

*2. The conclusion that there is a “rheostat-fashion” increase in Hcp1 is not correct, the data simply show additive effects of mutations. A rheostat effect requires demonstration of a gradual effect of change with changes in the amounts of the regulatory protein. Moreover, these effects were obtained using regulatory mutations with pleotropic effects, effecting other functions besides T6SS. This limitation should be acknowledged somewhere, either in the Results or Discussion.*

We have now removed the term “rheostat” and replace with “additive effect” to reflect the comment by this reviewer.

*3. Whereas it is clear how intermixing and expansion were determined, a few questions about the experimental setup remain. In most cases, why was not there an increase in the number of attacker cells (labeled with GFP) since the prey cells could not kill them? The exception is Figure 2B where the growth of killer cells at the edge is obvious.*

It is unclear what the reviewer’s comment is addressing. The example chosen from figure 2 is an example where we do not have T6SS competition but simply the same strain tagged either with Gfp or mCherry. So, in this case, there is no killer cells.

More generally, the intermixing measurement does not incorporate information about how much area is occupied by either of the strains, the only thing it suggests is how homogenous or segregated are the bacterial populations that are present. The overall size of a community is determined by the genetic background, setup parameters and growth conditions. Whether T6SS competition takes place or there is no competition at all has little to no impact on how large the colonies end up being. Mostly community size, growth time, motility, EPS, moisture and most of all nutrient limitation is what determines how big the colonies will end up being overall. If there is T6SS killing, attacker bacteria in green will be relatively more abundant. If the overall colony size remains unchanged and there is less prey, more of the space will be taken up by attacker. The size of community is fixed, and outcome is binary, if there is less prey there will be more attacker. This is the assumption that we rely on for measurements of relative prey abundance shown in the new supplementary figures S8 and S9 Figs that we have included in this new version of the paper to provide some specific quantification.

*4. The growth of prey cells, as shown by the formation of red sectors in the colony images, is surprising, since they should be killed by the attackers. Did the authors determine the ratio of viable cells (attackers and prey) in each colony? What are the surviving prey cells?*

This is an appropriate comment but one should take into account that clonal protection may come into play, so that prey cells are no longer in contact with attacker cells and can expand, and that could also be linked to the presence of for example dead preys surrounding these sectors. This is not something unusual and is documented in several other studies.

Furthermore, one main reason why some prey remains in specific sectors is likely due to the random placement of the bacteria in the inoculum spot. If prey bacteria happen to be on the edge of the spot and are surrounded only by kin cells, there is no reason for these to not expand. This is what the section about intermixing is about, and Fig5D/F illustrates this nicely. Simply trough chance, there will be some bacteria on the edge of the spot that will be shielded from attack, this is more common in low densities where fewer inter-population contacts are formed. Once the segments are established, the kin cells on the edge of the segment will form a protective barrier. As competitors are isogenic, neither grows faster, so there is no way for the attacker of consistently overtaking the prey population and removing established bacteria at this scale is unlikely. Another point that is relevant both here and for quantification is that between biological repeats where competition is effective, sometimes a single fragment will form, sometimes multiple small ones, other times none. A very precise quantification of the exact ratio in the outer region does not seem biologically meaningful. Attacker may take up 95% of the outer region or 100%, difference being only about the variation between repeats. With 3 repeats this does not seem like a meaningful difference to measure (see the new supplementary figure S8 Fig). We have also added a new supplementary figure S9 Fig to address numerical quantification of relative outer colony area occupied by prey and attacker bacteria.

*5. The figure legend for S5 Figure is insufficient. What are the two lines and three shaded areas? Why this Figure is also labeled Figure 2-Supplement 2?*

Yes the reviewer is correct and the figure was mislabelled. The legend of the figure has also been modified. As for the lines they are mean relative fluorescence intensity for each of the channels. The shaded area - error bars that indicate signal variance. For panel B - line indicates relative mean intermixing at given distance from colony centre, this is an index and no associated error is calculated

*6. The relative potency of the various toxins was assessed systematically throughout the paper as was the contribution of different mutations, using colony images. When assessing the relative effect of a mutation or potency of a toxin, some form of quantification is required, including appropriate statistical evaluation of the significance.*

Yes we agreed that some quantification is needed and have already addressed this in answers to previous comments.

To briefly add here we could consider the comment as a request for significance testing between competition outcomes in presence of T6SS+ and T6SS- attacker, averaging multiple biological repeats and significance testing the difference between the sets.

As shown in the new supplementary figure S8 Fig, the population distribution within a macrocolony is not uniform, hence a simple single number summary and any attempt to describe information from the image in a single number would discard most of the information that this assay provides.

One can measure the area (or fraction) of the colony occupied by prey bacteria, but this would ignore the most important aspect of this, that competition outcomes are distinct in the area where the population is established and upon further expansion. Therefore analysis has to distinguish the colony internal region and growing front.

The supplementary Fig 8 shows that the main competitive impact is observable in the colony outer region. Here we have also added the new supplementary figure S9 Fig, where relative prey abundance in the outer colony region is measured and we employ T-test to assess whether relative prey abundance is significantly impacted in presence of T6SS+ and T6SS- attackers.

*The quantification of intermixing in one experiment is shown in the S5B Fig. Therefore, it should be possible to evaluate and compare the relative Intermixing Indexes.*

The intermixing index is not indicative of the overall competitive outcome. This describes how bacteria that are present are distributed, but does not reflect relative abundance of populations. A very simple example would be that the outer region is split exactly in half, one large segment of green and one of red, each of the bacteria occupies 50% of the area, but they are not mixed at all. The measure shows how segmented the prey and attacker populations are, but the number of segments does not always directly relate to competition outcomes.

*These data should be shown, since it is hard to evaluate statements, such as “barely restricted” (line 183), “ restricted in growth” (line 184) or referring to the “most potent” toxins (lines 187,191 & 192) and “drastically improve” the impact of toxins (line 190).*

For this comment in particular and the text reference given here by the reviewer, it is important to say that we are not pointing at intermixing but rather at prey expansion as can be seen when looking exclusively at the red channel and comparing competition with a T6SS proficient attacker or a T6SS deficient attacker. In other words it means that the prey is no longer able to spatially expand beyond the point of inoculum region. It also means that “very high” T6SS activity, or potent toxin, would be required to impact prey growth to the extent where toxin sensitive bacteria are unable to grow even in the inoculum region.

These various sections of the text have thus been modified to clarify our statements, most of these being visually self-explanatory, but which are also now supported by quantification data, as described above and supported by the new supplementary figures S8 and S9 Figs.
